# The Projection-Specific Noradrenergic Modulation of Perseverative Spatial Behavior in Adult Male Rats

**DOI:** 10.1523/ENEURO.0063-24.2024

**Published:** 2024-08-15

**Authors:** Anna Kabanova, Leonid Fedorov, Oxana Eschenko

**Affiliations:** Department of Physiology of Cognitive Processes, Max Planck Institute for Biological Cybernetics, 72076 Tübingen, Germany

**Keywords:** anterior cingulate cortex, behavioral flexibility, executive function, norepinephrine, reward contingency, spatial learning

## Abstract

Adaptive behavior relies on efficient cognitive control. The anterior cingulate cortex (ACC) is a key node within the executive prefrontal network. The reciprocal connectivity between the locus ceruleus (LC) and ACC is thought to support behavioral reorganization triggered by the detection of an unexpected change. We transduced LC neurons with either excitatory or inhibitory chemogenetic receptors in adult male rats and trained rats on a spatial task. Subsequently, we altered LC activity and confronted rats with an unexpected change of reward locations. In a new spatial context, rats with decreased noradrenaline (NA) in the ACC entered unbaited maze arms more persistently which was indicative of perseveration. In contrast, the suppression of the global NA transmission reduced perseveration. Neither chemogenetic manipulation nor inactivation of the ACC by muscimol affected the rate of learning, possibly due to partial virus transduction of the LC neurons and/or the compensatory engagement of other prefrontal regions. Importantly, we observed behavioral deficits in rats with LC damage caused by virus injection. The latter finding highlights the importance of careful histological assessment of virus-transduced brain tissue as inadvertent damage of the targeted cell population due to virus neurotoxicity or other factors might cause unwanted side effects. Although the specific role of ACC in the flexibility of spatial behavior has not been convincingly demonstrated, our results support the beneficial role of noradrenergic transmission for an optimal function of the ACC. Overall, our findings suggest the LC exerts the projection-specific modulation of neural circuits mediating the flexibility of spatial behavior.

## Significance Statement

The reciprocal connectivity between the anterior cingulate cortex (ACC) and locus ceruleus (LC) implies a functional role for noradrenergic modulation in the ACC. The ACC and LC activation by unexpected events is thought to facilitate behavioral reorganization. The role of the LC→ACC pathway in spatial cognition remains largely unexplored. The ACC and LC connectivity with the hippocampal and parahippocampal regions provides an anatomical substrate for their involvement in the flexibility of spatial behavior. Using a chemogenetic approach, we found that activity suppression of the ACC-projecting LC neurons increased perseveration, while the suppression of the entire LC nucleus had the opposite effect. Our findings provide further evidence for the selective modulation of functionally distinct neural circuits via projection-specific LC neuron populations.

## Introduction

The brainstem noradrenergic nucleus locus ceruleus (LC) has been implicated in a variety of cognitive functions underlying adaptive behavior (Aston-Jones and Cohen, 2005; Sara, 2009). Unexpected events trigger the phasic discharge of LC neurons, and the associated simultaneous release of noradrenaline (NA) in spatially diffuse afferents has been suggested to facilitate neural network reorganization (Bouret and Sara, 2005; Sara and Bouret, 2012; Grella et al., 2021). Indeed, a positive correlation has been recently demonstrated between LC activity and the rate of behavioral switching; moreover, optogenetic LC activation accelerated task switching and improved task performance in mice (McBurney-Lin et al., 2022). The reciprocal connectivity between the LC and prefrontal cortex (PFC) is thought to support behavioral reorganization after the detection of an unexpected change (Bouret and Sara, 2004; Cerpa et al., 2023). The PFC is critical for adaptive behavior in a dynamic environment (Miller and Cohen, 2001). The role of descending prefrontal projections to the LC has been emphasized in regulating the balance between exploratory and exploitative behavior (Aston-Jones and Cohen, 2005; Kane et al., 2017; Cope et al., 2019).

The reciprocal connectivity between the anterior cingulate cortex (ACC) and the LC (Lewis et al., 1979; Morrison et al., 1979; Gompf et al., 2010; Chandler and Waterhouse, 2012; Koga et al., 2020) implies a functional role for noradrenergic modulation in the ACC (Tait et al., 2007; McGaughy et al., 2008; Gompf et al., 2010; Tervo et al., 2014; Koga et al., 2020). Temporal coupling between neuronal activity in the ACC and LC has been shown in nonhuman primates (Joshi and Gold, 2022). Optogenetic activation of the LC→ACC projection facilitated behavioral response to sensory stimulation in mice (Koga et al., 2020). Numerous studies of the ACC function in primates and humans concluded that the ACC contributes to cognitive control by integrating diverse inputs and downstream signaling for adjusting behavior to ongoing demands (Rushworth and Behrens, 2008; Heilbronner and Hayden, 2016; Monosov et al., 2020; Brockett and Roesch, 2021). The correlates of ACC activity with different components of cognitive control such as conflict detection, uncertainty estimation, or action selection have been also demonstrated in rodents (Walton et al., 2002; Haddon and Killcross, 2006; Tervo et al., 2014). The activation of ACC neurons by surprise or by detection of a change in the environment has been suggested to trigger an update of the internal cognitive model and to drive the exploration of alternatives (Hayden et al., 2011; Tervo et al., 2014). This view is supported by the switch-predictive firing of the ACC neurons (Sarafyazd and Jazayeri, 2019; Kane et al., 2022) and the deficit in behavioral switching in the ACC-lesioned animals (Brockett et al., 2020).

Remarkably, the role of ACC in cognitive control and behavioral flexibility has been predominantly established in humans and animal models using nonspatial behavioral paradigms, while the ACC contribution in spatial context received much less attention. The ACC connectivity with the parahippocampal regions provides an anatomical substrate for the ACC signaling to the network supporting spatial behavior (Jones and Witter, 2007). However, very limited experimental evidence has supported the involvement of the ACC in spatial cognition. Earlier studies found a behavioral deficit in spatial task acquisition, reversal learning, or spatial working memory in ACC-lesioned rats (Markowska et al., 1989; Seamans et al., 1995; Warburton et al., 1998; Dias and Aggleton, 2000; Amemiya et al., 2014), while other studies revealed no impairment (Neave et al., 1994; Ragozzino et al., 1998; Ragozzino and Kesner, 1998). A recent study in rats identified the ACC as a part of an extended brain network that is selectively activated during the acquisition of a new reward contingency rule on a T-maze (Oberto et al., 2023). The existing studies leave the question open whether NA release in the ACC promotes adaptive behavior in a spatial context (Amemiya et al., 2014; Xiang et al., 2019).

In the present study, we addressed this question by chemogenetic alteration of the NA transmission under conditions of uncertainty requiring the engagement of multiple components of cognitive control such as surprise/error detection, response inhibition, decision-making, strategy selection, and planning. We predicted that the ACC and LC activation by an unexpected change in the environment would facilitate behavioral reorganization. Contrary to our prediction, enhanced NA transmission did not affect behavioral flexibility. The inhibition of the ACC-projecting LC neurons increased perseveration errors, while suppression of the entire LC nucleus had the opposite effect. The behavioral flexibility was also impaired in rats with LC damage inadvertently caused by virus injection; the latter presents a serious concern for the functional assessment of the effects of chemogenetic manipulation of LC-NA neurons in wild-type rats. Overall, our results suggest that the LC-NA system exerts the projection-specific modulation of neural circuits mediating the flexibility of spatial behavior.

## Materials and Methods

### Animals

Fifty-four adult male Sprague Dawley rats (Charles River Laboratories) weighing 250–300 g at the beginning of the experiment were used. All experimental procedures were approved (study protocols KY01-19G and KY04-19G) by the local ethical commission (§15 TierSchG) and conducted in full compliance with the guidelines of the EU Directive on the protection of animals used for scientific purposes (2010/63/EU). Animals were maintained on 12 h light/dark cycles, with food and water *ad libitum* except for the duration of the learning experiment. The room temperature and humidity were kept at 20–23°C and 40–60%, respectively. All animals were group housed, except for a 1 week postsurgery recovery when the animals were single housed to facilitate wound healing. Cages were enriched with paper nesting material, cardboard tunnels, and small wooden objects.

### Viral transduction of noradrenergic neurons

To express the excitatory or inhibitory designer receptor exclusively activated by designer drugs (DREADDs) in the membrane of noradrenergic neurons, we used canine adenovirus type 2 (CAV2) based vector encoding a gene cassette for expression of a mutated human Gq-coupled M3 (hM3Dq) or Gi-coupled (hM4Di) muscarinic receptors under the control of synthetic PRSx8 promoter. Both vectors (CAV2-PRSx8-hM3Dq-mCherry and CAV2-PRSx8-hM4Di-HA-Tag) were provided by the Plateforme de Vectorologie de Montpellier, PVM, France. Previous studies have successfully used the CAV2-PRSx 8 viral construct to express opsins or DREADDs on the LC neurons (Hirschberg et al., 2017; Hayat et al., 2020; Stevens et al., 2021; Cerpa et al., 2023).

**Figure 1.**
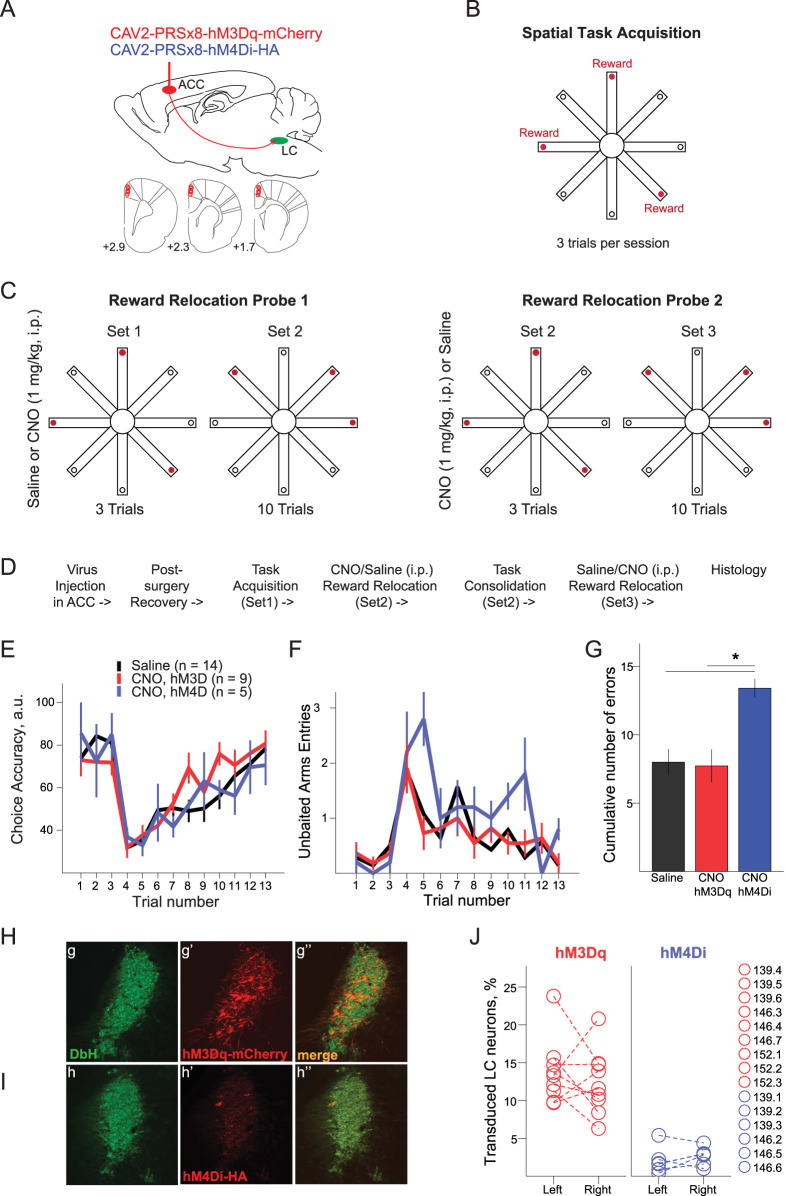
The effects of chemogenetic alternation of the noradrenergic transmission in the ACC on flexibility of spatial behavior. ***A***, The schematics of the virus injections in the ACC (top) and reconstruction of the injection sites (bottom). ***B***, The spatial memory task on an eight-arm radial maze. Rats were trained to retrieve three rewards from fixed locations at the end of maze arms during seven daily sessions (3 trials per day) or until the criterion performance (Extended Data Fig. 1-1). ***C***, The behavioral flexibility test. The Reward Relocation Probe was performed on the next day after reaching the criterion. At least 30 min before the maze session, randomly assigned rats received either CNO (1 mg/kg, i.p.) or saline. Three trials with “known” reward locations (Set1) followed by 10 trials with “unknown” baited maze arms (Set2). The test was repeated in a subsequent session (Set3) under different drug conditions. There was no difference in the learning rate for Set2 and Set3 (Extended Data Fig. 1-2). ***D***, The experimental timeline. ***E–G***, The choice accuracy (***E***) and perseveration errors (***F***, ***G***) during the Reward Relocation Probe. The data after saline injection were combined regardless of the DREADD type and Reward Set (2 or 3) and served as control. There were no significant between-group differences in the number of maze arm entries, trial time, or entries to previously baited arms (Extended Data Fig. 1-3). ***H***, ***I***, Representative sections showing immunostained LC neurons expressing DbH (***h***, ***i***), excitatory (***h’***), and inhibitory (***i’***) DREADDs and an overlay (***h’’***, ***i’’***). ***J***, The proportion of transduced LC neurons is shown for each injection case and each viral vector. Dashed lines connect data obtained from the same rat. *p < 0.05 (Bonferroni’s post hoc tests). The DREADD expression level was stable for up to 10 weeks after the virus injection (Extended Data Fig. 1-4). The ACC inactivation by muscimol did not affect the rate of learning (Extended Data Fig. 1-5).

The virus injections were performed under isoflurane anesthesia (Fig. 1*A*). The rat was anesthetized (5% induction, ∼1.2% maintenance) and fixed in a stereotaxic frame (David Kopf Instruments). The skull was exposed and craniotomies were made above the target regions. For retrograde transduction from the ACC, three injections of the virus suspension (500 nl, 1.5 × 10^12^ vp/ml) with a total volume of 1.5 μl (per hemisphere) were made using the following coordinates: AP: 1.5, 1.8, and 2.1 mm, L: ±0.8 mm, DV: 1.4 mm. For transduction of the entire LC, the initial LC coordinates (AP: −4.2 mm; L: ±1.2 mm from the bregma; Paxinos and Watson, 2005) were adjusted based on electrophysiological recording. The pipette was tilted 15° to the skull surface, and three injections of the viral suspension (333 nl of 1 × 10^12^ vp/ml) with a total volume of 1 μl (per hemisphere) were made along the dorsoventral axis in direct proximity to the LC core (−6.2, −5.9, and −5.6 mm from the brain surface). The virus was injected via a glass capillary pipette (40 μm tip) at a rate of 50 nl/min using a programmable nanoliter injector Nanoject III (Drummond Scientific Company). After each injection, the pipette was kept in place for 5 min to minimize the upward flow of viral solution and slowly withdrawn. After the last injection, a two-component silicone gel (Kwik-Sil: World Precision Instruments) was applied to the craniotomy for brain protection and the skin above the exposed skull was sutured. Before awakening from anesthesia and during the postsurgery recovery period, the rat was treated with an antibiotic (Baytril; 5 × 5.0 mg/kg, s.c.) and analgesic (Rimadyl, Zoetis; 3 × 2.5 mg/kg, s.c.). Rats were kept in their home cages with food and water *ad libitum* until the behavioral testing began.

10.1523/ENEURO.0063-24.2024.f1-1 Figure 1-1 Spatial task acquisition. Top and middle panels, Choice accuracy over the task acquisition period is shown for individual rats and different experimental groups. All rats were given 7 training sessions (3 trials per day); after that, training continued until reaching the learning criterion (> 70%) for at least one session to avoid overtraining. Choice accuracy was gradually increasing in all rats, albeit with inter-individual variability. Bottom panel, Choice accuracy is plotted for the last task acquisition session for all rats. Download Figure 1-1, TIF file.

**Figure 2.**
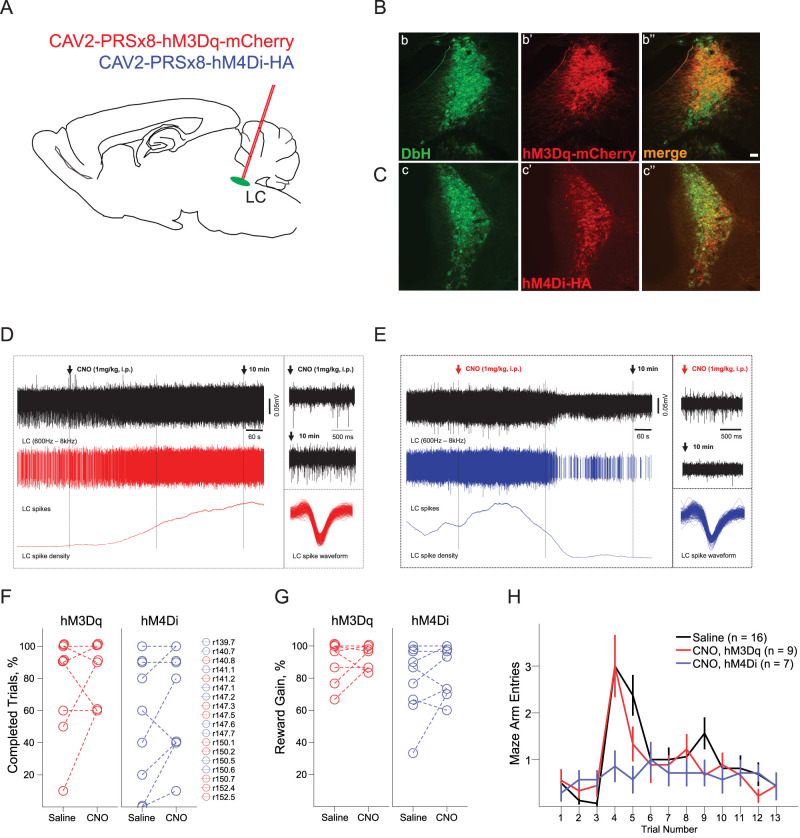
The effects of chemogenetic modulation of LC activity on flexibility of spatial behavior. ***A–C***, The virus transduction of LC-NE neurons. ***A***, The schematics of the virus injection in the LC area. ***B***, ***C***, Representative sections showing the LC-NE neurons expressing excitatory (***B***) and inhibitory (***C***) DREADDs. The proportion of virus-transduced LC neurons (Extended Data Fig. 2-1). ***D***, ***E***, Extracellular recordings of the LC spiking before and after CNO injection. Note a rapid and sustained modulation of the LC single units expressing hM3Dq (***D***) and hM4Di (***E***). ***F–H***, The rat performance on the Reward Relocation Probe. The percent of completed trials (***F***) and the reward gain (***G***) are plotted for hM3Dq- and hM4Di-expressing rats after saline and CNO (1 mg/kg, i.p.). Two rats failed the task performance after the reward relocation (Extended Data Fig. 2-2). ***H***, The number of maze arm entries before the first correct choice is plotted before (trials 1–3) and after (trials 4–13) the reward relocation. Note that the CNO-induced decrease in NE transmission resulted in fewer perseveration errors. There was no between-group difference in the rate of learning (Extended Data Fig. 2-3).

10.1523/ENEURO.0063-24.2024.f1-2 Figure 1-2 Rate of relearning during repeated reward relocations. All animals were administered both CNO and saline and were tested in successive sessions in a counterbalanced order. Choice accuracy improved equally across all trials during the first (Reward Set 2) and second (Reward Set 3) test sessions. Download Figure 1-2, TIF file.

**Figure 3.**
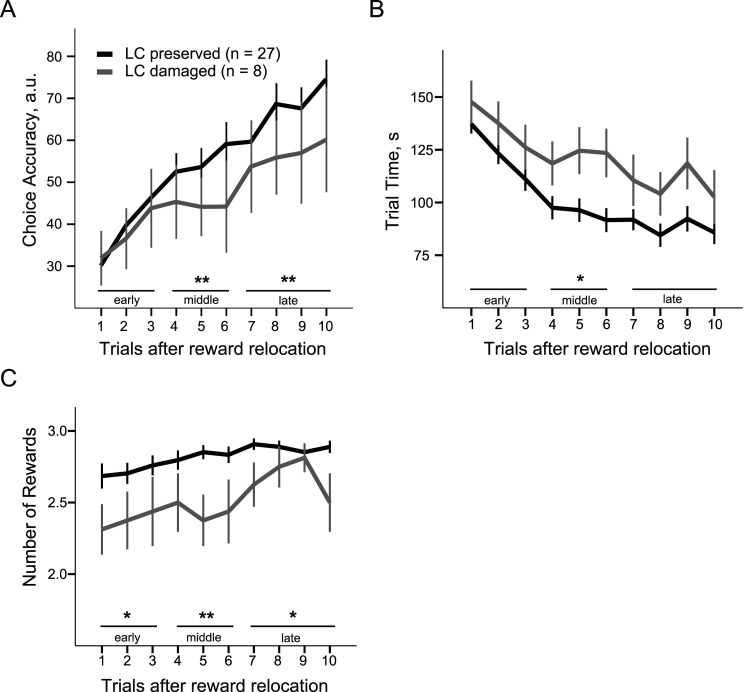
The impaired flexibility of spatial behavior in rats with neuronal loss in the LC. The choice accuracy (***A***), trial time (***B***), and reward gain (***C***) during the Reward Relocation Probe are plotted for the ACC- and LC-injected rats with preserved (>50%) and damaged (<50%) LC nuclei. Significant between-group differences for the choice accuracy (*F*_(1,101)_ = 9.76; *p* = 0.002), trial time (*F*_(1,101)_ = 4.75; *p* = 0.032), and reward gain (*F*_(1,101)_ = 10.34; *p* = 0.002) were indicative for overall less efficient learning in the rats with LC damage. The number of working and reference memory errors (Extended Data Fig. 3-1). The post hoc comparisons were performed for early (1–3), middle (4–6), and late (7–10) trials. *p < 0.05 and **p < 0.01 (one-way ANOVA).

10.1523/ENEURO.0063-24.2024.f1-3 Figure 1-3 The rat behavior during the Reward Relocation Probe. The total number of maze arm entries (A), trial time (B), and entries to previously baited arms (C) are plotted for all trials and experimental groups. The data after saline injection were combined for hM3Dq- and hM4Di-expressing rats. All rats showed equal food-motivated behavior as reflected by the number of maze arm entries in each trial (A). The trial time increased (B) due to rats’ visiting previous reward locations (C). The entries to unbaited maze arms gradually reduced. There was no significant trial x group interaction for any of the variables (Multivariate Pillai's Trace Test; entries: F(18,40) = 1.12, p = 0.372; time: F (6.1,163.9) = 0.86, p = 0.595; unbaited: F(18,40) = 0.78, p = 0.713). Download Figure 1-3, TIF file.

**Figure 4.**
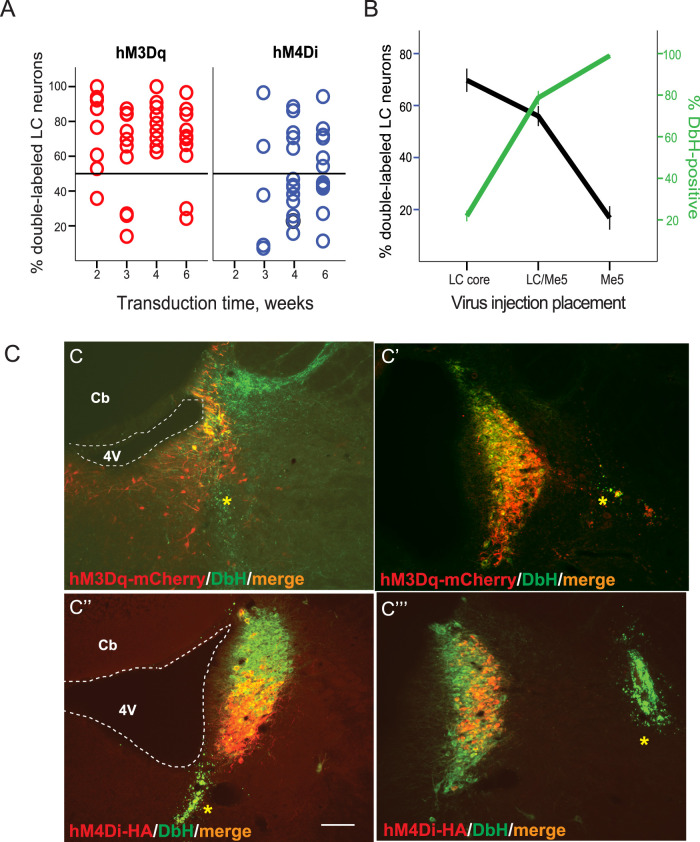
The efficiency of the CAV2-based vectors for DREADD transduction in the LC-NE neurons. ***A***, The proportion of double-labeled LC neurons for each case is plotted as a function of the virus transduction time. ***B***, The dependence of the transduction rate and LC-NE integrity on the virus injection placement. ***C***, Examples of case-specific transduction of the LC nucleus. The coronal sections at the LC plane were stained with DbH (green), mCherry (red), or HA-Tag (red), and an overlay (orange). The virus injection sites are labeled with green fluorescent beads (yellow asterisks). The representative examples with the injection made in the LC core resulting in severe neuronal loss (***C***); the injection placed ∼100 µm lateral to the LC core resulting in sufficient virus spreading (***C’***); the injection made medial-ventral to the LC core resulting in ventral infection of LC neurons (***C’’***); the virus delivered >200 µm lateral to the LC core resulting in partial virus transduction in the LC-NE neurons (***C’’’***). 4v, fourth ventricle; cb, cerebellum. Scale bar: 100 µm.

10.1523/ENEURO.0063-24.2024.f1-4 Figure 1-4 DREADD expression level in the ACC-projecting LC neurons did not decay for up to 10 weeks after the virus injection for either viral construct. Download Figure 1-4, TIF file.

10.1523/ENEURO.0063-24.2024.f1-5 Figure 1-5 The ACC transient inactivation by muscimol did not affect the rate of relearning during the Reward Relocation Probe. A total of 10 muscimol (n = 10 rats) and 12 saline (n = 12 rats) injections were included in the analysis. Download Figure 1-5, TIF file.

### Immunohistochemistry and quantification of virus transduction

To quantify the virus construct efficiency after the behavioral testing, the rats were administered a lethal dose of sodium pentobarbital (100 mg/kg i.p.; Narcoren, Merial) and then perfused transcardially with 0.9% buffered saline followed by 4% paraformaldehyde in 0.1 M phosphate buffer (PBS), pH 7.4. Brains were extracted and postfixed in the same fixative overnight and then transferred to 30% sucrose solution in PBS at 4°C for at least 72 h before sectioning at 40 µm thickness on a sliding microtome (Microm HM 440E).

Free-floating sections were rinsed three times (5 min) in 0.2% Triton X-100 prepared in PBS (PBST) and subsequently incubated in a blocking buffer containing 10% normal donkey serum (NDS) in PBST for 1 h at room temperature, followed by incubation with primary antibodies at 4°C overnight. The slices were washed three times in 0.2% PBST and incubated with secondary antibodies at room temperature for 2 h. To label the nuclei, all slices were counterstained with Hoechst, followed by three rinses with 0.2% PBST. Slides were then mounted with Aqua-Polymount mounting medium (Polysciences) and covered with coverslips. Immunofluorescence labeling was observed and acquired under the fluorescent microscope (AxioVision, Carl Zeiss). Images were acquired using a 20× objective (AxioVision, Carl Zeiss) with structured illumination and analyzed by using ImageJ software. The DREADD expression in the LC-NA neurons was evident by the mCherry or HA-Tag labeling of DbH-expressing LC neurons. mCherry- or HA-Tag-positive cells coexpressing DbH were counted in the LC and normalized for the total number of DbH^+^ cells counted. To assess the LC-NE integrity, DbH-positive cells were counted within the LC borders in the virus-transduced site and normalized to the average of DbH-positive LC-NA neurons in intact rats.

### Electrophysiological validation of the CAV2-PRSx8 constructs for modulation of noradrenergic neuron activity

The functional validity of viral vectors was confirmed by in vivo electrophysiology in anesthetized rats receiving intra-LC virus injections. Briefly, at the end of behavioral testing, the rat was anesthetized with urethane and fixed in the stereotaxic frame and the skull exposed. A single tungsten electrode (FHC) was placed in the LC, and spiking activity was recorded before and after CNO injection (1 mg/kg, i.p.) for at least 1 h.

### Behavioral procedures

To allow sufficient virus spreading, for the LC-transduced rats, the learning experiment began immediately after a 1 week postsurgery recovery and 2–3 weeks after the virus injection for the ACC-transduced rats (Fig. 1*B*). All rats were first familiarized with a reward (chocolate milk) in their home cages and accustomed to gentle handling by an experimenter. At least 24 h before the learning experiment began, rats were put on a food-controlled regimen (∼15 g of food pellets per rat per day). The restricted food schedule was maintained for the entire duration of the learning experiment. Rat's weight was controlled daily to ensure that it was not below 85% of their *ad libitum* body weight. Animal behavior was tested on a custom-made eight-arm radial maze. The maze consisted of eight alleys (66 cm long × 10 cm wide) extended from a central round platform (30 cm diameter). A small (1 cm diameter) round pit was located at the end of each maze alley for food reward (a few drops of chocolate milk). Two large visual cues were hung on the black curtains surrounding the maze. An elevated stand (20 × 30 cm) placed at a fixed corner of the room was used for keeping the rat during an intertrial interval (ITI) and also served as a 3D extramaze cue. The experimental room was dimly lit. After placing a rat in the maze, the experimenter quickly left the room and monitored the rat's behavior through a video camera until the trial ended. Rat training took place during the dark phase of the light/dark cycle and at the same time for each rat. During one to three daily 20 min habituation sessions, rat behavior on the maze was shaped to retrieve at least three rewards at the end of any maze alley within a 5 min cutoff time. As soon as the rat expressed reward-oriented behavior and readily collected rewards, the task acquisition began. During the task acquisition sessions, three out of eight randomly selected maze arms were baited (Fig. 1*B*). At the beginning of each trial, the rat was placed on the central platform and allowed 5 min to collect rewards. During the ITI, the maze surface was wiped and reward pits were rebaited. The reward locations were randomly assigned for each rat and maintained fixed for the entire task acquisition period consisting of seven daily sessions (three trials per session). In a few cases, additional training was required to reach the learning criterion (Extended Data Fig. 1-1). This behavioral design minimized the task performance based on procedural (striatal-dependent) strategy, while maximizing the load on spatial memory and therefore making the task hippocampal dependent.

After reaching the criterion of at least 70% correct choices and 5–7 weeks after virus injection, we tested the flexibility of spatial behavior (Fig. 1*C*, Reward Relocation Probe). Specifically, after three standard trials (Reward Set1), three new baited arms were assigned (Reward Set2), and the rat was allowed 10 trials to adjust its behavior to a new spatial context. In rare cases when the rat did not perform to the criterion during the first three trials (Set1), the Reward Relocation Probe was postponed to the next day, and this session was considered as additional training. On the following day after the Reward Relocation Probe, rats were given a consolidation session (10 trials) with the same configuration of baited arms (Set2). The second reward relocation test (Set3) was given after reaching the asymptote performance with Set2. This experimental design allowed within-animal comparison of the behavioral effects of chemogenetic modulation (Fig. 1*D*).

### Intracerebral injections in the ACC

For intrabrain injections, a subset of virus-transduced rats (*n* = 25) received chronically implanted cannulas in the ACC. The surgery took place after the chemogenetic experiment and ∼5–7 weeks postvirus injection. Surgery was performed under isoflurane anesthesia following similar procedures as described above for the virus injection. In a fully anesthetized rat, a midline incision was made, the skull was exposed, and craniotomies were made above the ACC (AP: 1.8 mm, L: ±0.8 mm). Two single guide cannulas (26-gauge, Plastics One) were stereotaxically placed above the ACC dorsal border (DV: 1.4 mm). Four anchoring screws were fixed at the skull edges and the implant was secured with dental cement (Paladur, Heraeus Kulzer). A dummy cannula was inserted to prevent clogging.

After at least a 5 d postsurgery recovery, rats were habituated to the intrabrain injection procedure. A dummy cannula was removed and replaced with the inner cannula (28 gauge, Plastics One) that extended 1 mm below the guide cannula. The inner cannula was connected to a 5 μl Hamilton syringe via a polyethylene tube filled with the drug. The syringe was driven by an infusion pump (UMP3, World Precision Instruments) controlling the injection of a total volume of 0.5 μl at a rate of 0.25 μl/min. At least 1 min was allowed for drug diffusion and to avoid backflow of the drug cocktail up the cannula track.

### Drugs

All drug solutions were prepared on the day of injection. The clozapine-*N*-oxide (CNO, Enzo Life Sciences), a potent agonist of DREADDs, was dissolved in sterile water (Ampuwa, Fresenius) to a concentration of 1 mg/ml and administered systemically (1 mg/kg, i.p.) at least 30 min before behavioral testing. The muscimol powder (Sigma-Aldrich) was diluted in saline at a final concentration of 0.1 μg/μl. A volume of 0.5 μl was injected bilaterally in the ACC. The muscimol concentration and injection volume were selected based on earlier studies (Tervo et al., 2014; Wartman et al., 2014; Carrillo et al., 2019). According to published data (Martin, 1991; Edeline et al., 2002), we estimated the radial spreading of muscimol of at least 1.0 mm within 30 min following the injection and the effect persists for ∼2 h.

### Experimental design and statistical analysis

The spatial behavior of virus-injected rats (*n* = 36) was tested over multiple daily sessions. Each animal was tested after saline and CNO during separate Reward Relocation Probe sessions. After completion of the chemogenetic experiment, a subset of rats (*n* = 25) was used for the ACC inactivation experiment. A separate cohort of virus-injected rats (*n* = 18) was used for histology only. The following behavioral variables were analyzed: (1) the total number of maze arms visited; an entry to the maze arm was considered when a rat left the central platform with all four limbs; (2) trial time, the time required to collect three rewards; (3) choice accuracy, a ratio between entries into the baited and all maze arms; (4) working memory errors were estimated by the number of re-entries into the maze arms; and (5) reference memory errors were estimated by the number of entries into unbaited maze arms. The behavioral variables were submitted to the repeated-measures ANOVA with the trial and drug condition as repeated factors. The Greenhouse–Geisser correction was applied when the sphericity assumption was violated. The variables derived from the histological material were submitted to a one-way ANOVA. The statistical significance (*α* value) was set at *p* = 0.05. The IBM SPSS Statistics (v.22) was used for statistical analysis. The key sources are summarized in the Extended Data Table 1-1. The original data are available on request.

10.1523/ENEURO.0063-24.2024.t1-1 Table 1-1 Key resources. Download Table 1-1, DOCX file.

## Results

### Suppression of NA transmission in the ACC resulted in perseverative behavior

To assess the role of noradrenergic transmission in the ACC for flexibility of spatial behavior, we retrogradely transduced the ACC-projecting LC neurons in adult wild-type male rats with either excitatory (hM3Dq, *n* = 9 rats) or inhibitory (hM4Di; *n* = 6 rats) DREADDs (Fig. 1*A*) and subsequently trained rats on a spatial memory task (Fig. 1*B*). Typically, rats reached the choice accuracy of >70% after seven daily training sessions, while a few rats required additional training (Extended Data Fig. 1-1). One rat (case 139.3) failed to learn the task after 14 training sessions and was excluded from further behavioral testing. In the last task acquisition session, the choice accuracy was 83.0 ± 5.2 (*n* = 9) and 89.2 ± 3.2 (*n* = 5) for hM3Dq- and hM4Di-expressing rats (one-way ANOVA; *F*_(1,13)_ = 0.70; *p* = 0.42).

The behavioral flexibility was tested on the Reward Relocation Probe (see Materials and Methods for details). Briefly, rats trained to retrieve rewards from three fixed maze arms were confronted with an unexpected change of baited maze arms and allowed to adjust their behavior to a new spatial context. The Reward Relocation Probe was performed on the next day after reaching the learning criterion and consisted of 3 trials with “known” reward locations (Set1) followed by 10 trials with “unknown” reward locations (Set2; Fig. 1*C*, left). At least 30 min before the Reward Relocation Probe, a subset of rats received CNO (1 mg/kg, i.p.) while others received saline. The rat assignment to the drug was random and blind to the rat trainer. On the following day, rats were given the Set2 “consolidation” session; in rare cases, additional training was required to reach the criterion performance before the Reward Relocation Probe (Set3) was repeated after a different drug (Fig. 1*C*, right). Figure 1*D* shows the experimental timeline. Importantly, the rate of learning for Set2 and Set3 was similar (Multivariate Pillai's Trace Test, trial × Set interaction: *F*_(9,18)_ = 0.87, *p* = 0.57; Extended Data Fig. 1-2). We next compared the rat behavior after activation (hM3Dq + CNO, *n* = 9) or inhibition (hM4Di + CNO, *n* = 5) of the ACC-projecting LC neurons with the combined data after saline injection serving as control (*n* = 14). Importantly, there was no between-group difference in the task performance before the reward relocation (Fig. 1*E*, trials 1–3). During the next 10 trials, most rats (12 out of 14) were able to retrieve all three rewards from new locations in the vast majority (>80%) of trials within the cutoff time, while the remaining two rats had a lower reward gain due to <80% of completed trials. Regardless of the differential reward gain, DREAAD type, or drug, all rats equally expressed food-motivated behavior by actively visiting maze arms (Extended Data Fig. 1-3*A*). Neither chemogenetic activation nor inhibition of the ACC-projecting LC neurons affected the rate of learning (Fig. 1*E*, trials 4–13). The repeated-measures ANOVA confirmed the behavioral change across trials (Multivariate Pillai's Trace Test; *F*_(9,19)_ = 13.71; *p* < 0.001) with no trial × group interaction (*F*_(18,40)_ = 0.85; *p* = 0.64). Expectedly, the time to collect rewards increased due to erroneous visits to previously baited maze arms (Extended Data Fig. 1-3*B,C*). None of the other variables including reference and working memory errors (see Materials and Methods) revealed the effect of DREADD activation (data not shown).

We then reasoned that the effectiveness of behavioral switching may be reflected by the number of entries into unbaited arms before the first correct choice is made. Since most of the errors were due to entering previously rewarded maze arms (Extended Data Fig. 1-3*C*), this behavior could be interpreted as perseveration. Indeed, rats with reduced NA transmission in the ACC entered nonbaited maze arms more persistently (*F*_(2,25)_ = 4.71; *p* = 0.018; Fig. 1*F*). Since the repeated-measures ANOVA revealed no significant trial × group interaction (*F*_(18,40)_ = 0.85; *p* = 0.64), we omitted the post hoc tests across trials. The cumulative number of perseveration errors significantly differed between groups (one-way ANOVA; *F*_(2,29)_ = 5.10; *p* = 0.013) with CNO-induced deficit in rats expressing hM4Di in ACC-projecting LC neurons (Fig. 1*G*). After completing the behavioral experiment, we quantified the retrograde transduction of the ACC-projecting LC neurons. Table 1 summarizes the results of the case-based quantitative immunohistochemistry analysis. Figure 1, *H* and *I*, shows the representative sections with DREADD-expressing DbH-positive LC neurons. The delivery of CAV2-PRSx8-hM3Dq-mCherry into ACC retrogradely labeled 13.1 ± 1.0% (range, 6.3–23.8%) of DbH-positive LC neurons (Fig. 1*H*), which was consistent with other studies reporting the proportion of prefrontal-projecting LC neurons (Chandler and Waterhouse, 2012; Hirschberg et al., 2017; Iqbal et al., 2023). The visualization of hM4Di-expressing LC neurons was less successful (2.3 ± 0.4% of DbH-positive LC neurons; range, 0.4–5.4%; Fig. 1*I*), possibly due to HA-Tag. The DREADD expression level of both types of chemoreceptors remained stable up to 10 weeks postinjection (Extended Data Fig. 1-4). Importantly, for both viral vectors the brain tissue within the LC area was well preserved, showed no signs of neurotoxicity, and showed high DbH expression level within the LC core (data not shown).

**Table 1.** Retrograde transduction efficiency of the ACC-projecting LC neurons

	Rat ID	TR time, weeks	% Transduced^a^	
			Left LC	Right LC
CAV2-PRSx8-hM3D-mCherry	139.4	8	14.7	14.8
	139.5	9	13.3	20.8
	139.6	9	9.7	11.6
	146.3	10	23.8	14.9
	146.4	10	9.8	13.9
	146.7	10	13.7	10.2
	152.1	7	11.3	6.3
	152.2	7	12.1	10.8
	152.3	7	15.7	8.4
CAV2-PRSx8-hM4D-HA	139.1	8	0.4	2.8
	139.2	9	5.4	4.4
	139.3	8	0.9	1.6
	146.2	10	1.7	2.9
	146.5	10	2.2	1.0
	146.6	10	1.6	3.0

a Percent DbH-positive LC neurons expressing DREADDs.

Thus, inhibition of the ACC-projecting LC-NE neurons resulted in perseverative behavior but did not affect the rate of learning. An unexpectedly small number of LC-NA neurons expressing hM4Di receptors (∼2%) could be attributed to HA-Tag visualization rather than the low transduction efficiency of the CAV2-PRSx8-hM4Di-HA-Tag. Contrary to our prediction, an enhanced NA transmission in the ACC did not affect the flexibility of spatial behavior. Although at least ∼13% of the ACC-projecting LC neurons were virus transduced, their activation by CNO did not change rat behavior; alternatively, the experimental design and behavioral variables analyzed were not sufficiently sensitive to reveal the behavioral effects.

### Transient inactivation of the ACC did not impair the flexibility of spatial behavior

Considering a rather subtle effect of inhibition and no effect of activation of the NA transmission in the ACC, we sought to validate if the ACC is critical for behavioral reorganization in a new spatial context. To this end, we implanted a total of 25 virus-transduced rats, with bilateral cannulas in the ACC. After postsurgical recovery, rats were retrained on the maze task until criterion and tested on the Reward Relocation Probe, as described in the Results section above (see Materials and Methods for details). Immediately before the Reward Relocation Probe, rats received either muscimol, a GABAA receptor agonist, or saline injection bilateral in the ACC. Five rats were excluded from the analysis due to premature implant loss, misplaced, or clogged cannulas, and one rat failed to perform the task; therefore, the data from 19 rats were included in statistical analysis. Most rats were tested on the Reward Relocation Probe (Set 3) after either saline (*n* = 9 rats) or muscimol (*n* = 7 rats), and three rats received both saline and muscimol injections on two consecutive sessions (Set 3 and 4). Thus, the resulting dataset included 10 muscimol (*n* = 10 rats) and 12 saline (*n* = 12 rats) injections. The ACC inactivation did not affect the rate of learning (trial × group: *F*_(9,12)_ = 1.57, *p* = 0.231; Extended Data Fig. 1-5). None of the other behavioral variables revealed the effect of ACC inactivation (data not shown). Although we did not detect the effect of ACC inactivation, our results do not rule out the ACC involvement in the behavioral reorganization triggered by an unexpected change in the environment. The lack of behavioral effects could be due to compensatory engagement of other prefrontal regions. Moreover, our findings support the beneficial role of NA transmission in the ACC for flexibility of spatial behavior.

### Effects of global modulation of NA transmission on the flexibility of spatial behavior

We next assumed that the NA transmission beyond the LC→ACC pathway might contribute to behavioral reorganization in a new spatial context. To this end, we used the CAV2-PRSx8-hM3Dq-mCherry (*n* = 10) or CAV2-PRSx8-hM4Di-HA-Tag (*n* = 11) to transduce the entire population of the LC-NA neurons (Fig. 2*A*) and tested rats’ behavioral flexibility after increased or decreased global NA transmission. Figure 2, *B* and *C*, shows the representative LC sections with virus-transduced DbH-positive LC neurons. The results of quantitative histological analysis for each case are summarized in Tables 2 and 3. In one rat, there was severe bilateral LC damage (case 139.8; Table 2), and in the other two rats, hM4Dq expression was completely lacking (cases 150.3 and 150.4; Table 3); therefore, these cases were excluded from the behavioral analysis. Overall, 72.1 ± 5.0 and 54.4 ± 5.5% of LC-NA neurons expressed hM3Dq and hM4Di, respectively, although with great within- and intersubject variability (Extended Data Fig. 2-1). The chemogenetic modulation of the virus-transduced LC neurons was additionally verified by in vivo electrophysiology (Fig. 2*D,E*).

10.1523/ENEURO.0063-24.2024.f2-1 Figure 2-1 The proportion of virus-transduced LC neurons in the left and right hemispheres. Download Figure 2-1, TIF file.

10.1523/ENEURO.0063-24.2024.f2-2 Figure 2-2 The number of maze arm entries (left) and the choice accuracy (right) during the Reward Relocation Probe. Note a robust increase in the number of maze arm entries after the reward relocation on trial 4, except for two rats showing low exploratory activity. The same two rats had the slowest relearning rate (poor switch) despite the good task performance before the reward relocation. The rat behavior did not depend on the drug injection. Download Figure 2-2, TIF file.

10.1523/ENEURO.0063-24.2024.f2-3 Figure 2-3 The rate of relearning in rats with enhanced (red) or decreased (blue) NE transmission. All animals were administered both CNO and saline and were tested in successive sessions in a counterbalanced order. The choice accuracy improved across trials equally in all groups (F (6.1,21) = 12.72, p < 0.001, Greenhouse-Geisser corrected; trial x group: F(12.2,44) = 0.62, p = 0.89). Download Figure 2-3, TIF file.

**Table 2.** Transduction efficiency of the CAV2-PRSx8-hM3Di-mCherry for LC-NE neurons

Rat ID	Virus transduction time, weeks	Transduced LC-NE, % (avg)^b^	Preserved LC-NE, % (avg)	Injection site	Injection placement	Transduced LC-NE, %	Preserved LC-NE, %
150.7^a^	4	94.1	21.1	Left LC	cLC (0 µm)	88.1	39.8
				Right LC	cLC (0 µm)	100.0	2.4
156.7	2	89.9	80.7	Right LC	LC/Me5 (<100 µm)	87.4	68.9
				Left LC	LC/Me5 (<100 µm)	92.3	92.5
136.1	3	85.9	10.3	Right LC	cLC (0 µm)	84.6	5.1
				Left LC	cLC (0 µm)	87.2	15.4
152.5^a^	6	85.8	8.05	Right LC	cLC (0 µm)	75.0	4.7
				Left LC	cLC (0 µm)	96.6	11.4
134.2	3	79.2	32.3	Right LC	cLC (0 µm)	74.3	39.8
				Left LC	cLC (0 µm)	84.1	24.8
147.3^a^	6	77.8	60.2	Left LC	LC/Me5 (<100 µm)	71.4	78.3
				Right LC	LC/Me5 (<100 µm)	84.1	42.1
141.2^a^	6	76.9	5.75	Left LC	cLC (0 µm)	66.7	2.4
				Right LC	cLC (0 µm)	87.0	9.1
150.2^a^	4	76.8	69.3	Right LC	LC/Me5 (<100 µm)	70.3	67.7
				Left LC	LC/Me5 (<100 µm)	83.3	70.9
140.8^a^	4	76.7	9.65	Left LC	cLC (0 µm)	62.5	6.3
				Right LC	cLC (0 µm)	90.9	13.0
147.4	4	76.7	26	Right LC	cLC (0 µm)	74.5	37.0
				Left LC	cLC (0 µm)	78.9	15.0
156.6	2	73.4	65.9	Left LC	LC/Me5 (<100 µm)	52.9	100.0
				Right LC	cLC (0 µm)	93.8	31.9
133.2	2	68.7	47.8	Left LC	ventral cLC (0 µm)	60.7	35.0
				Right LC	LC/Me5 (<100 µm)	76.6	60.6
103.1	2	67.9	31.7	Right LC	LC/Me5 (<100 µm)	35.8	52.8
				Left LC	cLC (0 µm)	100.0	10.6
133.1	3	64.7	79.7	Right LC	LC/Me5 (<100 µm)	59.6	59.4
				Left LC	LC/Me5 (<100 µm)	69.7	100.0
147.5^a^	6	50.0	67.2	Left LC	LC/Me5 (<100 µm)	29.9	77.6
				Right LC	LC/Me5 (<100 µm)	70.1	56.7
112.1	3	46.4	91.2	Right LC	Me5 (≤150 µm)	26.9	98.0
				Left LC	LC/Me5 (<100 µm)	65.9	84.3
152.4^a^	10	42.4	94.9	Left LC	Me5 (≤150 µm)	24.3	100.0
				Right LC	LC/Me5 (<100 µm)	60.5	89.8
150.1^a^	4	32.9	13.2	Right LC^c^	cLC (0 µm)	n/a	0.0
				Left LC	cLC (0 µm)	65.7	26.4
134.1	3	20.0	54.1	Right LC	LC/Me5 (<100 µm)	14.0	67.3
				Left LC	cLC (0 µm)	26.0	40.9
139.8^a^	6	n/a	1.4	Left LC^c^	cLC (0 µm)	n/a	2.8
				Right LC^c^	cLC (0 µm)	n/a	0.0

a Rats tested in the maze.

b Cases are sorted according to the average percent of transduced LC neurons.

c Cases excluded from quantitative histological analysis due to severe tissue damage in the LC area.

**Table 3.** Transduction efficiency of the CAV2-PRSx8-hM4Di-HA-Tag for LC-NE neurons

Rat ID	Virus transduction time, weeks	Transduced LC-NE, % (avg)^b^	Preserved LC-NE, % (avg)	Injection site	Injection placement	Transduced LC-NE, %	Preserved LC-NE, %
147.6^a^	4	85.3	24.0	Right LC	cLC (0 µm)	84.1	24.8
				Left LC	cLC (0 µm)	86.4	23.2
139.7^a^	6	83.0	60.1	Left LC	LC/Me5 (<100 µm)	73.7	100.0
				Right LC	cLC (0 µm)	92.2	20.1
129.1	6	56.1	51.4	Left LC	LC/Me5 (<100 µm)	42.1	67.3
				Right LC	cLC (0 µm)	70.0	35.4
147.1^a^	6	54.0	81.1	Right LC	LC/Me5 (<100 µm)	39.6	100.0
				Left LC	LC/Me5 (<100 µm)	68.4	62.2
147.7^a^	4	51.9	32.1	Right LC	cLC (0 µm)	41.2	20.1
				Left LC	cLC (0 µm)	62.5	44.1
147.2^a^	6	49.9	92.3	Right LC	LC/Me5 (<100 µm)	42.7	91.3
				Left LC	LC/Me5 (<100 µm)	57.0	93.3
156.2	3	49.6	60.8	Left LC	LC/Me5 (<100 µm)	35.6	74.0
				Right LC	LC/Me5 (<100 µm)	63.6	47.6
156.4	3	47.2	n/a	Left LC	LC/Me5 (<100 µm)	94.4	84.6
				Right LC^c^	v-m	n/a	n/a
150.6^a^	4	46.1	63.8	Left LC	LC/Me5 (≤100 µm)	23.6	100.0
				Right LC	cLC (0 µm)	68.6	27.6
140.7^a^	4	46.1	59.1	Left LC	Me5 (≤150 µm)	20.7	98.8
				Right LC	cLC (0 µm)	71.4	19.3
150.5^a^	4	42.5	99.8	Left LC	LC/Me5 (<100 µm)	40.2	100.0
				Right LC	LC/Me5 (<100 µm)	44.7	99.6
129.3	6	33.0	5.2	Left LC	cLC (0 µm)	25.0	1.6
				Right LC	cLC (0 µm)	40.9	8.7
141.1^a^	6	30.7	51.0	Right LC	cLC (0 µm)	9.2	55.9
				Left LC	LC/Me5 (<100 µm)	52.1	46.1
152.7	4	28.4	92.5	Left LC	LC/Me5 (<100 µm)	20.7	100.0
				Right LC	LC/Me5 (<100 µm)	36.1	85.0
152.6	4	22.8	62.8	Right LC	cLC (0 µm)	13.6	26.0
				Left LC	LC/Me5 (<100 µm)	32.0	99.6
136.2	3	6.0	99.0	Right LC	Me5 (≤150 µm)	5.1	100.0
				Left LC	Me5 (≤150 µm)	6.8	98.0
150.3^a^	4	0.0	98.8	Left LC^d^	Me5 (≤150 µm)	0.0	100.0
				Right LC^d^	Me5 (≤150 µm)	0.0	97.6
150.4^a^	4	0.0	100.0	Left LC^d^	Me5 (≤150 µm)	0.0	100.0
				Right LC^d^	Me5 (≤150 µm)	0.0	100.0
156.3	3	n/a	n/a	Left LC3	n/a	n/a	n/a
				Right LC^d^	n/a	n/a	n/a

a Rats tested in the maze.

b Cases are sorted according to the average percent of transduced LC neurons.

c No DbH expression in the LC area.

d No mCherry expression; cases marked with c and d were excluded from the quantitative histological analysis.

We next compared the rats’ behavior after the CNO-induced LC activation or inhibition and saline injections serving as control. First, there was a substantial variability in the rat behavior after the reward relocation. Namely, not all rats were able to find new reward locations in every trial (Fig. 2*F*); the latter resulted in different reward gains (Fig. 2*G*). However, the behavioral variability could be attributed to the interindividual differences and not to the chemogenetic modulation (one-way ANOVA, completed trials: *F*_(2,31)_ = 0.46, *p* = 0.63; reward gain: *F*_(2,31)_ = 0.57, *p* = 0.57). Besides, two hM4Dq-expressing rats had extremely low exploratory activity and failed to perform the task in a new spatial context (Extended Data Fig. 2-2) and therefore were excluded from further analysis. The comparison of perseveration errors (entries to unbaited arms before the first correct choice) revealed a striking between-group difference (*F*_(2,29)_ = 4.50; *p* = 0.014) as the rats with reduced NA transmission show less perseveration (Bonferroni’s post hoc tests). Moreover, there was a significant trial × group interaction (*F*_(9.9,44)_ = 1.78; *p* = 0.028; Fig. 2*H*) due to the main difference in the first two trials after the reward relocation. Nevertheless, the rate of learning was similar across groups (choice accuracy: *F*_(6.1,21)_ = 12.72, *p* < 0.001; Greenhouse–Geisser corrected; trial × group: *F*_(12.2,44)_ = 0.62, *p* = 0.89; Extended Data Fig. 2-3). None of the other behavioral variables (see Materials and Methods) revealed a significant between-group difference (data not shown). Thus, the reduction of global NA transmission appeared to be beneficial for the reorganization of spatial behavior, yet did not speed up learning. The latter effect contrasted enhanced perseveration caused by inhibition of NA transmission in the ACC and likely mediated by a different neural circuit. Rather surprisingly, there was no effect of global LC activation, which could be attributed, for example, to partial LC activation or the null effect due to activation of the competing functional networks.

### Loss of LC-NA neurons impaired flexibility of spatial behavior

The overall number of chemogenetically modulated LC-NA neurons depended not only on the virus transduction efficiency and receptor expression but also on the LC integrity. Since in some cases, the LC integrity was seriously compromised, we combined all rats regardless of the injection site (ACC and LC) and type of DREADDs according to the LC integrity index and compared their behavior on the Reward Relocation Probe. The LC integrity index averaged at 74.2 ± 2.8% in rats with >50% of LC neurons preserved (*n* = 27 rats) and at 13.1 ± 2.2% in rats with damaged LC (*n* = 8 rats). The rats with LC damage were less efficient in adjusting their behavior to a new spatial context (choice accuracy: *F*_(1,101)_ = 9.76, *p* = 0.002; Fig. 3*A*). The repeated-measures ANOVA revealed a significant effect of the trial (*F*_(8,93)_ = 25.23, *p* < 0.001; Greenhouse–Geisser corrected), but no trial × group interaction (*F*_(8,93)_ = 1.49; *p* = 0.157); therefore, we did not perform post hoc tests on individual trials. However, there was a significant trial × group interaction when we grouped the trials into early (1–3), middle (4–6), and late (7–10) after the reward relocation (*F*_(2,100)_ = 3.96; *p* = 0.22). The post hoc comparisons revealed that the choice accuracy was significantly lower in the middle and late trials confirming the slower learning rate in the LC-lesioned rats (Fig. 3*A*). Further analysis showed that the LC-lesioned rats had difficulty in finding new reward locations as indicated by an overall longer trial time (*F*_(1,101)_ = 4.75; *p* = 0.032). There was no significant trial × group interaction (*F*_(7.2,93)_ = 0.74; *p* = 0.644), but the middle trials were significantly longer (Fig. 3*B*). Moreover, rats with LC damage were unable to collect all three rewards within a trial cutoff time (*F*_(1,101)_ = 10.34; *p* = 0.002; Fig. 3*C*). There was no significant trial × group interaction (*F*_(6.2,93)_ = 1.39; *p* = 0.213; Greenhouse–Geisser corrected), yet the reward gain differed significantly at each phase of the session (Fig. 3*C*). Finally, the behavioral deficit in the LC-lesioned rats could not be attributed to spatial memory errors. The results pattern was rather inconsistent as the LC-lesioned rats made fewer WM and RM errors in the early trials and more RM errors in the late trials (Extended Data Fig. 3-1). Overall, there was no between-group difference for either error type (WM: *F*_(1,101)_ = 2.63, *p* = 0.108; RM: *F*_(1,101)_ = 0.00, *p* = 0.985). Thus, multiple factors or their cumulative effect likely resulted in a less efficient behavioral reorganization in rats with <50% of the LC nucleus preserved.

10.1523/ENEURO.0063-24.2024.f3-1 Figure 3-1 The number of working (left) and reference (right) memory errors during the Reward Relocation Probe is shown for the rats with preserved (n = 27) and damaged (n = 8) LC. ** - p < 0.05 (pna-way ANOVA). Download Figure 3-1, TIF file.

### CAV2-PRS8-based DREADD expression variability in the LC neuron population

For better characterization of the efficiency of virus transduction in the LC, we injected an additional cohort of rats either with CAV2-PRSx8-hM3Dq-mCherry (*n* = 10 rats) or CAV2-hM4Dq-HA-Tag (*n* = 8 rats) bilaterally into the LC area and for histology analysis combined both cohorts with the transduction time spanning from 2 to 6 weeks. Three out of 40 injections (*n* = 20 rats) of the CAV2-PRSx8-hM3Dq-mCherry and 7 out of 38 injections (*n* = 19 rats) of the CAV2-hM4Dq-HA-Tag were invalid due to the absence of immunostaining or poor tissue quality. Substantial intersubject variability in the transduction of LC-NA neurons remained in a larger dataset (Fig. 4*A*). On average, 69.5 ± 3.7% (min/max: 14.0/100%) of LC-NA neurons expressed the excitatory DREADDs and 47.4 ± 4.6% (min/max: 5.1/94.4%) of the LC-NA neurons expressed the inhibitory DREADDs. The proportion of DREADDs-expressing LC neurons did not depend on the duration of transduction time (Fig. 4*A*). A more detailed histological examination revealed that the intersubject variability could be attributed to the injection placement relative to the LC core (Fig. 4*B*; Tables 2, 3). Specifically, targeting the LC dendritic zone extending laterally to the LC core (up to 100 μm) was the most successful as it resulted in a very effective transduction of the LC-NA neurons while preserving the population of LC-NA neurons (Fig. 4*C*). The injections along the LC dorsoventral axis and medial to the LC core primarily transduced the ventral population of LC-NA neurons (Fig. 4*C*). The injections placed lateral to the LC core within the trigeminal tract mainly transduced the lateral LC population (Fig. 3*C*).

In contrast to the retrograde transduction approach, in the case of virus injection in the LC area, the tissue in the injection site was frequently seriously compromised, particularly when the virus was delivered close to the LC core (Fig. 3*C*). We evaluated the LC integrity as the proportion of preserved DbH-positive neurons compared with the neuronal count in the intact LC (see Materials and Methods). The LC integrity index varied from 0 to 100% averaging at 38.8 ± 8.2 and 62.6 ± 8.2% for the hM3Dq-mCherry and M4Dq-HA-Tag, respectively. In the case of hM3Dq, more than half of the LC nucleus was preserved in 17 out of 40 cases (42.5% of injections); the remaining injections caused more severe neuron loss leaving from zero to 42.1% of NA neurons (Table 2). In the case of hM4Di, at least half of LC-NA neurons were preserved in 22 out of 36 cases (61.1%); in the remaining cases, the LC integrity index varied from 1.6 to 46.1% (Table 3). Notably, the high transduction efficiency reversely correlated with the number of preserved DbH-positive LC neurons (Fig. 4*B*). Thus, the neuronal loss and/or tissue damage inadvertently caused by virus injection may present a serious concern for the functional assessment of the effects of chemogenetic manipulation of LC-NA neurons in wild-type rats.

## Discussion

We designed the present study considering extensive evidence about the involvement of both the ACC and LC in driving adaptive behavior triggered by a change in the environment. As most studies have used nonspatial paradigms, we specifically focused on whether LC→ACC interaction is required for spatial behavior. To this end, we expressed inhibitory or excitatory DREADDs in the LC neurons projecting to the ACC and trained rats to retrieve rewards from fixed locations in the eight-arm radial maze. To test behavioral flexibility, we presented rats with an unexpected change in reward location while DREADDs were activated by CNO. The choice accuracy in a new spatial context expectedly decreased due to competition between the established behavior and exploration triggered by reward uncertainty. We found that decreased NA transmission in the ACC caused perseveration, while the suppression of global LC activity had the opposite effect. However, chemogenetic modulation of perseverative behavior did not affect the rate of learning. Notably, we observed behavioral deficits in rats with compromised LC integrity caused by virus injection.

### Task- and context-dependent involvement of the LC→ACC pathway in spatial cognition

Successful task performance in a new spatial context required the engagement of multiple components of cognitive control and behavioral flexibility. Namely, a sudden change of reward locations was unavoidably associated with a surprise/error detection, which was expected to drive exploratory behavior not only to find new reward locations but also to verify if the task rule remained the same or if a different strategy had to be adapted. Both, the ACC and LC, are activated in situations of uncertainty when attention, behavioral optimization, and/or exploration are engaged (Sara et al., 1995; Aston-Jones and Cohen, 2005; Yu and Dayan, 2005; Rushworth and Behrens, 2008; Sara and Bouret, 2012; Monosov et al., 2020; Brockett and Roesch, 2021). Previous studies linked the cerulear–prefrontal interactions to interrupting the ongoing activity and promoting exploratory behavior (Sara and Bouret, 2012; Tervo et al., 2014; Kane et al., 2017). Optimal task performance required inhibiting entry to previously baited maze arms, searching and memorizing new reward locations, and avoiding repeated entry to the same maze arm. We expected that under these conditions, the ACC and LC activation might facilitate learning and updating the internal representation of the current spatial context.

In our experiments, most (but not all) rats were able to find all new reward locations already in the first trial and gradually optimize their behavior over successive trials. Inhibition of the ACC-projecting LC neurons caused perseverative behavior, supporting the role of NA in the ACC for inhibitory control after error detection. This result was consistent with a documented surge of prefrontal NA following the detection of a change in reward prediction in rats during the performance of a spatial working memory task on a T-maze (Rossetti and Carboni, 2005) or a visuospatial attention task (Dalley et al., 2001). Furthermore, reduced NA transmission in the ACC has been shown to increase neophobia and impaired spatial memory in aged rats (Collier et al., 2004). Silencing of noradrenergic inputs in the ventrolateral orbitofrontal cortex, but not in the prelimbic cortex, impaired updating identity-specific action–outcome associations when environmental conditions change (Cerpa et al., 2023). At the same time, a heightened perseveration did not affect the rate of learning. It is possible that the spatial task and/or the behavioral flexibility test used in the present study might have not sufficiently engaged the ACC and LC to allow for revealing more pronounced behavioral effects. Alternatively, an unaltered NA transmission within other prefrontal subregions compensated for the deficit of NA in the ACC. Surprisingly, we did not detect any effects of increased NA in the ACC, albeit both beneficial and deleterious effects could be expected depending on the magnitude of NA release. In a nonspatial context, DREADD-mediated stimulation of LC terminals within the medial PFC improved strategy set-shifting in rats through faster adaptation to a new rule (Cope et al., 2019). Although the underlying circuit is currently unknown, the observed dissociation supports the projection-specific effects of NA and differential modulation by NA of neural circuits controlling adaptive behavior.

In contrast to NE depletion in the ACC, suppressing the activity of the entire LC reduced perseveration. One possibility is that reduced NA signaling in the amygdala resulted in weaker anxiety and, therefore, faster behavioral switch. However, neither LC activation nor inhibition affected the learning rate. Various behavioral deficits were observed after pharmacological or chemogenetic inhibition of NA transmission (Khakpour-Taleghani et al., 2009; Amemiya et al., 2014; Jahn et al., 2018; Hamlett et al., 2020), but the effects appear to be task specific. For example, NA-depleted transgenic mice were deficient when tested on the attentional set-shifting but spatial task (Isingrini et al., 2023). Other studies have demonstrated that in a nonspatial context, a higher LC activity predicted faster behavioral switching to a new rule (Kane et al., 2017; Rorabaugh et al., 2017; McBurney-Lin et al., 2022). The timing of NA release might be important, and since we manipulated the LC tonic activity, the task-related fluctuations of NA release could be more essential. Yet, we observed pronounced behavioral deficits in rats with LC damage caused by virus injection. Finally, our results concerning adult male rats and gender, as well as age-dependent effects of NA depletion on different aspects of spatial learning and memory and behavioral flexibility, have been reported (Langlais et al., 1993; Benloucif et al., 1995; Collier et al., 2004; Rorabaugh et al., 2017; Gheidi et al., 2023).

Finally, although the ACC role in spatial cognition was not the main focus of the present study, we found no evidence that the ACC activity is essential for behavioral adaptation to a new spatial context. Our present findings are in agreement with earlier work showing that ACC lesions did not impair performance on spatial tasks (Neave et al., 1994; Ragozzino et al., 1998), nor affected the strategy switch from spatial to a visual-cued version of the cheeseboard task (Ragozzino et al., 1999b). Another study in monkeys reported weak or no effects of ACC lesions when task switching required behavioral optimization without the task rule change (Rushworth et al., 2003). Remarkably, an unaltered cognitive control has been documented in patients with dorsal ACC damage; the latter observation is consistent with the view that seemingly normal behavioral adjustments have been achieved via an alternate network (Fellows and Farah, 2005). One study reported perseverative behavior in ACC-lesioned rats when they were tested on a radial maze task requiring holding information about visited locations in short-term memory (Seamans et al., 1995). Consistently, the ACC activation has been shown during the acquisition of a new reward contingency rule on a T-maze (Oberto et al., 2023). The discrepancy in experimental findings might be due to the fact that the involvement of different subregions of the PFC is task and context specific (Neave et al., 1994; Rushworth et al., 2003; Rich and Shapiro, 2007; Monosov et al., 2020). For example, inactivation of the medial PFC did not impair the intramodal (place–place) strategy switch (reversal learning) on a T-maze, but the cross-strategy (place–response) switch (Ragozzino et al., 1999a). Our results are the most consistent with the view that the ACC mainly monitors the inner state, external context, and/or behavioral performance but is not essential for triggering the neural and/or behavioral switch (Amiez et al., 2005; Wallis and Rich, 2011). Alternatively, a compensatory engagement of other prefrontal subregions might have accounted for the lack of behavioral deficit of ACC inactivation (Ragozzino et al., 1999a; Tait et al., 2007; McGaughy et al., 2008; Oualian and Gisquet-Verrier, 2010; Oberto et al., 2023).

### Heterogeneous DREADD expression in the LC and compromised LC integrity

Besides methodological aspects related to the sensitivity of the behavioral paradigm used in the present study, we encountered several important challenges to be mindful of when using chemogenetics. In recent years, the use of conventional lesion and reversible inactivation methodology has been replaced by a new methodology permitting more selective modulation of neural circuits. The discussion that follows addresses some of the pitfalls of chemogenetic targeting small brain regions like the LC. Key caveats include careful histological evaluation on a case-by-case basis and interpretation of behavioral results based on correct estimation of the proportion of modulated cells relative to the entire target cell population.

Our quantitative histological assessment, while confirming the efficiency of the CAV2 virus and the PRSx8 promoter to transduce NA neuronal cell bodies and axonal terminals (Chillon and Kremer, 2001; Soudais et al., 2001), identified a discrepancy in the DREADD expression. Specifically, we reported successful retrograde labeling of the LC→ACC neurons using the CAV2-PRSx8-hM3Dq-mCherry as the number of hM3Dq-expressing LC-NA neurons (∼13%) was comparable to anatomical studies (Chandler and Waterhouse, 2012; Hirschberg et al., 2017; Iqbal et al., 2023). In contrast, the expression of hM4Di-HA-Tag in ACC-projecting LC-NA neurons was substantially lower (∼2%). A similar discrepancy between the hM3Dq-mCherry and hM4Di-HA-Tag expression levels was observed after direct injections in the LC area. One of the possible reasons for lower efficiency could be the tag detection method. Unlike the visible expression of fluorescent proteins in the cell cytoplasm, HA-Tag allows the detailed spatial localization of the hM4Di receptors in the cell only with immunohistochemical analysis and tag-specific antibodies. The affinity of antibodies for small tags can be low, resulting in insufficient detection when the protein of interest is weakly expressed. Furthermore, peptide epitopes are not stably expressed in cells without fusion to a scaffold protein (Viswanathan et al., 2015). Therefore, the use of peptide tags fused with fluorescent protein can increase the detection of receptor expression (Zhu et al., 2016) and therefore provide a more accurate estimate of the number of transduced cells. Interestingly, some of the studies showed that HA-Tag restricts the DREADD expression to the plasma membrane, while mCherry reporter gene expression is confined in intracellular space (Vazey and Aston-Jones, 2014; Galvan et al., 2019). Thus, due to the possibility of hM4Dq-HA trafficking to the plasma membrane and dendrites, counting the number of LC-NA cell bodies expressing hM4Dq-HA (Vazey and Aston-Jones, 2014) might not provide the most accurate estimate of DREADD expression.

In addition to challenges with the detection of reporter expression, the virus transduction efficiency and neuronal loss due to cytotoxicity might significantly affect the total number of CNO-modulated LC neurons. Regardless of the viral vector, injection within ∼100 µm from the LC core resulted in the most profound LC-NA virus transduction and minimal neuronal loss, whereas injection within 50 µm or directly into the LC core led to partial or complete cell loss. The injections within the LC dendritic zone (∼200 µm from the LC core), while preserving most of the LC cell bodies, resulted in only partial transfection, largely due to the retrograde capability of CAV2 (Zussy et al., 2016). Interestingly, other studies suggested low cytotoxicity of the CAV2 based on examination of cell morphology, axonal arborization, or ultrastructure of CAV2-transduced neurons (Simão et al., 2016; Li et al., 2018). In our study, we report the direct correlation between the proximity of the injection site, transduction rate, and neuronal cell loss. Moreover, the sensitivity of LC-NA neurons to damage can aggravate the activation of microglia and cause neuroinflammation leading to reduced or absent secretion of NA and neurodegeneration (Alba-Delgado et al., 2016; Cao et al., 2019). Here, we reported behavioral deficit in rats with severely compromised LC integrity (<50% preserved), possibly due to decreased basal and evoked NA release after the LC depletions greater than 50% (Abercrombie and Zigmond, 1989). Notably, though the LC-NA system can neurochemically compensate for lesions of 75–90% of the LC-NA neurons by increasing LC firing rates (Chiodo et al., 1983), it remains to be established if this mechanism is engaged in the case of chemogenetic modulation of the LC activity.

### Concluding remarks

Our present study further supported the role of the LC-NA system in the flexibility of spatial behavior. Specifically, we found differential effects of local (in ACC) and global suppression of NE transmission on perseverative behavior under uncertainty in a spatial context. Our findings are coherent with an emerging view that LC exerts selective modulation of functionally distinct neural circuits. The retrograde transduction of projection-specific LC neurons proves to be an important tool for further dissecting the mechanisms of heterogeneous neuromodulation. That being said, our present results are consistent with the generally accepted view that even the use of the most efficient vectors for introducing DREADD does not lead to 100% penetrance of the target cell population. The chemogenetic modulation of the entire LC-NA population may rarely be achieved and therefore should not be assumed when interpreting the study outcomes. Our findings highlight the importance of careful histological assessment as inadvertent damage of the targeted cell population due to virus neurotoxicity or other factors might cause unwanted side effects. Nevertheless, better LC transduction in combination with an optimized behavioral paradigm will likely permit the revealing of more pronounced and specific behavioral effects.

### Limitations of the study

It is important to note that the present study has some limitations. First, while the lack of behavioral effects of ACC inactivation does not rule out the role of ACC in spatial cognition, the specific role of ACC in the flexibility of spatial behavior has not been convincingly demonstrated. Future studies are likely to reveal behavioral effects of ACC inactivation using other spatial tasks and clarify several open questions raised by the present study such as the role of noradrenergic modulation in other prefrontal subregions or their compensatory engagement. Second, our results highlighted challenges in using viral tools to target and manipulate small nuclei in wild-type rodents. To overcome the limitation of viral tropism and delivery proximity, the use of transgenic animals and Cre- or Flp-dependent constructs enable unprecedented access to cells. However, the use of genetically modified animal models for behavioral studies must be with caution due to possible developmental alternations. Thus, there is a need for the development of more efficient and selective viral capsids for targeting small nuclei in wild-type animals. Lastly, technical challenges resulted in a small sample size in some of the experiments, and these results should be replicated to be considered reliable.

## Synthesis

Reviewing Editor: Laura Bradfield, University of Technology Sydney Decisions are customarily a result of the Reviewing Editor and the peer reviewers coming together and discussing their recommendations until a consensus is reached. When revisions are invited, a fact-based synthesis statement explaining their decision and outlining what is needed to prepare a revision will be listed below. The following reviewer(s) agreed to reveal their identity: Etienne Coutureau, Michael Ragozzino.

Thank you for submitting the revised version of your manuscript regarding the projection-specific noradrenergic modulation of perseverative spatial behavioural adult male rats. Upon re-review, however, the reviewers and myself still found significant issues with the manuscript that are yet to be resolved. Reviewer 1 notes that without an experiment demonstrating the role of anterior cingulate cortex (ACC) in spatial cognition, some of the claims of the manuscript are not well supported. Upon consultation with the reviewers, my suggestion is that the manuscript be re-written in a way that reflects the results presented. Specifically, I suggest that the reason that the retrograde manipulations in Figure 1 alter spatial cognition could be due to the transfection of LC neurons that, although they are clearly projecting to ACC, are also projecting to other regions. Therefore, it could just be the LC neurons themselves that have a role in the task (via projections to other regions) rather than the LC-->ACC pathway. If the authors do not wish to complete an experiment showing a role for ACC in this task, then my suggestion is that the manuscript be re-written in a way that emphasises the role of LC in spatial cognition, and that this role could be via ACC or could be via other projections.

Alternatively, if the authors do decide to complete an experiment in which they show a role for ACC, and this will take longer than two months, please let send me an email and let me know.

I would also like to highlight some other issues to address. First, the sample size in the experimental groups is quite small (n = 5) in Figure 1, and I think it should be acknowledged in text somewhere that the effect should be replicated before being considered reliable. Second, please address reviewer 1's remaining concerns about histology. And finally, in line with the final suggestion of reviewer 1, please carefully edit the manuscript to ensure all necessary details are present and correct.

Reviewer #1

In this revised manuscript, the authors have addressed some of the concerns I raised in the initial submission. However, unfortunately, some of my major concerns remain. They are listed below.

The first concern was about behaviour. I keep on considering that a logical first step would have been to run an initial experiment showing that the ACC is critical to correctly perform the task. The reversible inactivation experiment performed here is not particularly convincing given that it involved a second surgery in non-naïve animals. My second concern about histology has been only partially addressed. I keep considering that the differential expression of HM3Dq and HM4Di, due to different measurement is problematic. The authors have now included more details about the experimental timeline, inclusion/exclusion criteria, and also sample size for each experiment but some confusion remains (e.g. rationale of using different transduction times, rationale of using a separate cohort of animals, repeated confusion between HM4Di and HM3Dq in the text and figure captions...).

Reviewer #2

The authors have adequately addressed all of the major and minor concerns from the original submission.

## Author Response

eN-NRS-0063-24R1: The projection-specific noradrenergic modulation of perseverative spatial behavior in adult male rats Synthesis Statement for Author (Required):

Thank you for submitting the revised version of your manuscript regarding the projection-specific noradrenergic modulation of perseverative spatial behavior in adult male rats. Upon re-review, however, the reviewers and myself still found significant issues with the manuscript that are yet to be resolved. In particular, Reviewer 1 notes that without an experiment demonstrating the role of anterior cingulate cortex (ACC) in spatial cognition, some of the claims of the manuscript are not well supported. After consultation with the reviewers, my suggestion is that the manuscript be re-written in a way that reflects the results presented. Specifically, I suggest that the reason that the retrograde manipulations in Figure 1 alter spatial cognition could be due to the transfection of LC neurons that, although they are clearly projecting to ACC, are also projecting to other regions. Therefore, it could just be the LC neurons themselves that have a role in the task (via projections to other regions) rather than the LC-->ACC pathway. If the authors do not wish to complete an experiment showing a role for ACC in this task, then my suggestion is that the manuscript be re-written in a way that emphasises the role of LC in spatial cognition, and that this role could be via ACC or could be via other projections.

Alternatively, if the authors do decide to complete an experiment in which they show a role for ACC, and this will take longer than two months, please let send me an email and let me know.

I would also like to highlight some other issues to address. First, the sample size in the experimental groups is quite small (n = 5) in Figure 1, and I think it should be acknowledged in text somewhere that the effect should be replicated before being considered reliable. Second, please address reviewer 1's remaining concerns about histology. And finally, in line with the final suggestion of reviewer 1, please carefully edit the manuscript to ensure all necessary details are present and correct.

Response: We thank the Editor and Reviewers for positive evaluation of our revised manuscript and constructive suggestions. First, we do agree that an experiment demonstrating the role of anterior cingulate cortex (ACC) in spatial cognition would be complementary, albeit it goes beyond the focus of our present study on the role of noradrenergic modulation in the ACC in the flexibility of spatial behavior. Based on the vast literature, we hypothesized that the LC-ACC interaction is required for behavioral reorganization in a new spatial context. We designed the behavioral flexibility test to include the ACC-mediated components of cognitive control such as surprise/error detection, response inhibition, decision-making, strategy selection, and planning.

Unfortunately, given the current regulations in Germany, the approval of application for new experiments might take up to 1 year; therefore, collecting new data demonstrating the role of ACC in spatial cognition appears not feasible within at least 1-2 years.

In planning our experiments, we complied with 3R principle and tried to minimize the number of animals. Please note that a second surgery, as reimplantation of the ACC cannulas in our case, is not that uncommon, and is used when appropriate.

Following your suggestion, we completely restructured the Introduction and the Significance Statement to downscale the focus on the ACC and its role in spatial cognition. Instead, we highlighted the role of NA transmission in the ACC. We revised the manuscript to emphasize the role of LC in spatial cognition and to imply that the observed behavioral effect might be mediated by the collaterals of ACC-projecting LC neurons to other targets.

We have addressed the most important concerns in a new section: Limitations of the Study.

Limitations of the Study It is important to note that the present study has some limitations. First, while the lack of behavioral effects of ACC inactivation does not rule out the role of ACC in spatial cognition, the specific role of ACC in the flexibility of spatial behavior has not been convincingly demonstrated. Future studies are likely to reveal behavioral effects of ACC inactivation using other spatial tasks and clarify several open questions raised by the present study such as the role of noradrenergic modulation in other prefrontal subregions or their compensatory engagement. Second, our results highlighted challenges in using viral tools to target and manipulate small nuclei in wild-type rodents. To overcome the limitation of viral tropism and delivery proximity, the use of transgenic animals and Cre- or Flp-dependent constructs enable unprecedented access to cells. However, the use of genetically modified animal models for behavioral studies must be with caution due to possible developmental alternations. Thus, there is a need for the development of more efficient and selective viral capsids for targeting small nuclei in wild-type animals. Lastly, technical challenges resulted in a small sample size in some of the experiments and these results should be replicated to be considered reliable.

Reviewer #1 In this revised manuscript, the authors have addressed some of the concerns I raised in the initial submission. However, unfortunately, some of my major concerns remain. They are listed below.

The first concern was about behaviour. I keep on considering that a logical first step would have been to run an initial experiment showing that the ACC is critical to correctly perform the task. The reversible inactivation experiment performed here is not particularly convincing given that it involved a second surgery in non-naïve animals. My second concern about histology has been only partially addressed. I keep considering that the differential expression of HM3Dq and HM4Di, due to different measurement is problematic. The authors have now included more details about the experimental timeline, inclusion/exclusion criteria, and also sample size for each experiment but some confusion remains (e.g. rationale of using different transduction times, rationale of using a separate cohort of animals, repeated confusion between HM4Di and HM3Dq in the text and figure captions...).

Response: We do agree that the experiment demonstrating the role of ACC in spatial cognition would be complementary, albeit it goes beyond the focus of our present study on the role of noradrenergic modulation in the ACC in the flexibility of spatial behavior. Based on the vast literature, we hypothesized that the LC-ACC interaction is required for behavioral reorganization in a new spatial context. We designed the behavioral flexibility test to include the ACC-mediated components of cognitive control such as surprise/error detection, response inhibition, decision-making, strategy selection, and planning. Last but not least, in planning our experiments we complied with 3R principle and tried to minimize the number of animals. Please note that a second surgery, as reimplantation of the ACC cannulas in our case, is not that uncommon, and is used when appropriate.

We do agree that differential expression of HM3Dq and HM4Di might be problematic. However, despite the low expression of HM4Di-HA in LC neurons assessed via immunohistology, our new results on the effect of inhibition of the ACC-projecting LC neurons in combination with convincing electrophysiology results suggest that the HM4Di were fully functional. Besides, in the case of the direct injections in LC, the transduction efficiency was more comparable.

To comply with the German animal research regulations, we had to minimize the number of animals enrolled in the behavioral experiments and their potential suffering due to food and water deprivation by using a cohort of animals for histological analysis only.

We thank the reviewer for concerns regarding different virus transduction time. Based on the histological results, we determined the optimal virus spreading, with a minimum virus transduction time of 1 week for direct LC injection and 4 weeks for retrograde labeling from the ACC. After the minimal transduction time, the animals were submitted to behavioral experiment, therefore the actual transduction time in each animal dependent on the individual learning rates.

We revised the manuscript text to describe the rationale for each experiment, added more details, and thoroughly checked the text to avoid confusing descriptions.

We have addressed the most important concerns in a new section: Limitations of the Study.

Reviewer #2 The authors have adequately addressed all of the major and minor concerns from the original submission.
