# Supplementary figures and images for: The Projection-Specific Noradrenergic Modulation of Perseverative Spatial Behavior in Adult Male Rats
Source: eNeuro. 2024 Aug 15;11(8):ENEURO.0063-24.2024. doi: 10.1523/ENEURO.0063-24.2024 (PMC11334950; doi:10.1523/ENEURO.0063-24.2024)

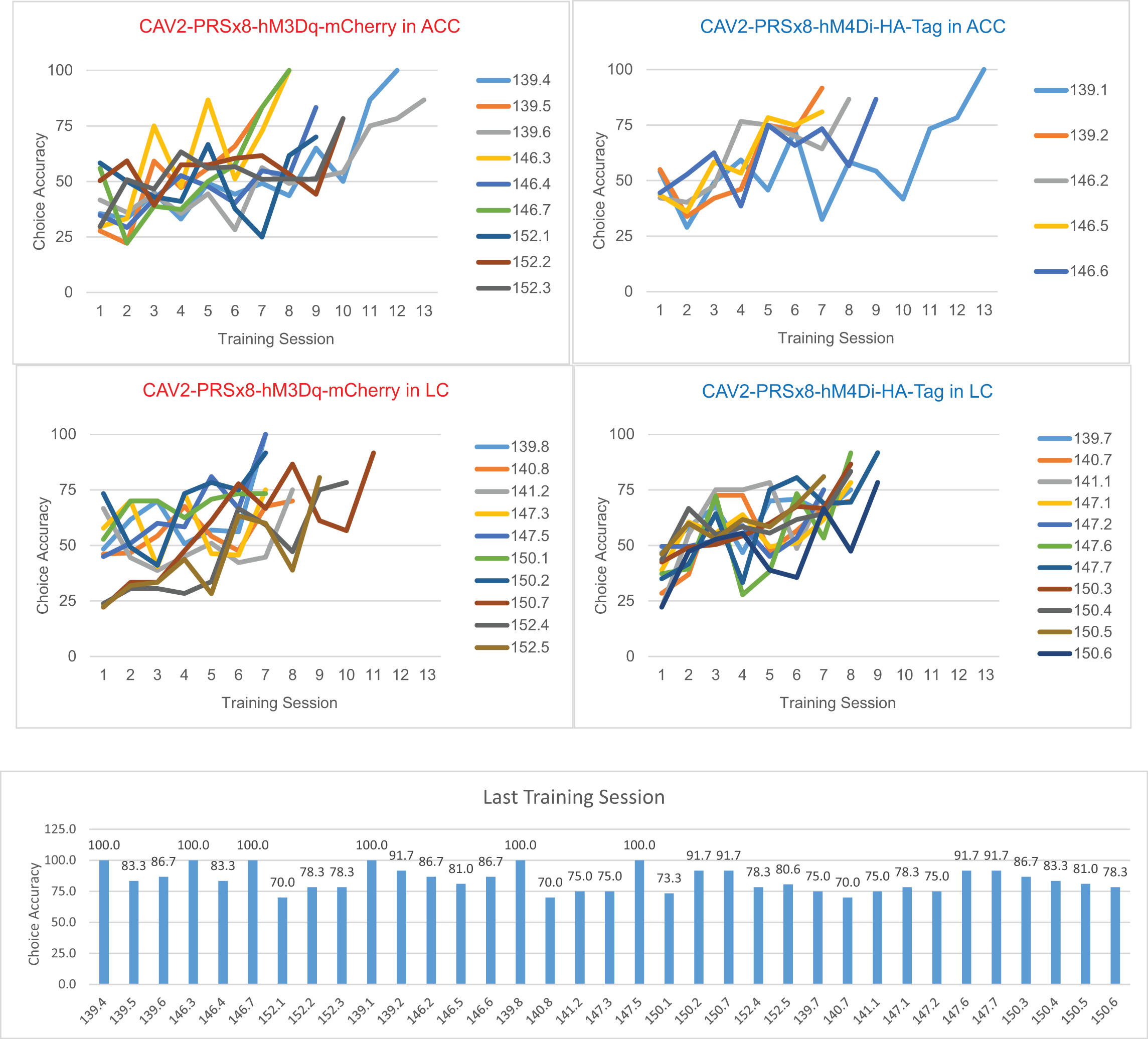

Supplement: Figure 1-1 — Spatial task acquisition. Top and middle panels, Choice accuracy over the task acquisition period is shown for individual rats and different experimental groups. All rats were given 7 training sessions (3 trials per day); after that, training continued until reaching the learning criterion (> 70%) for at least one session to avoid overtraining. Choice accuracy was gradually increasing in all rats, albeit with inter-individual variability. Bottom panel, Choice accuracy is plotted for the last task acquisition session for all rats. Download Figure 1-1, TIF file. [file eneuro-11-ENEURO.0063-24.2024-s003.tif]

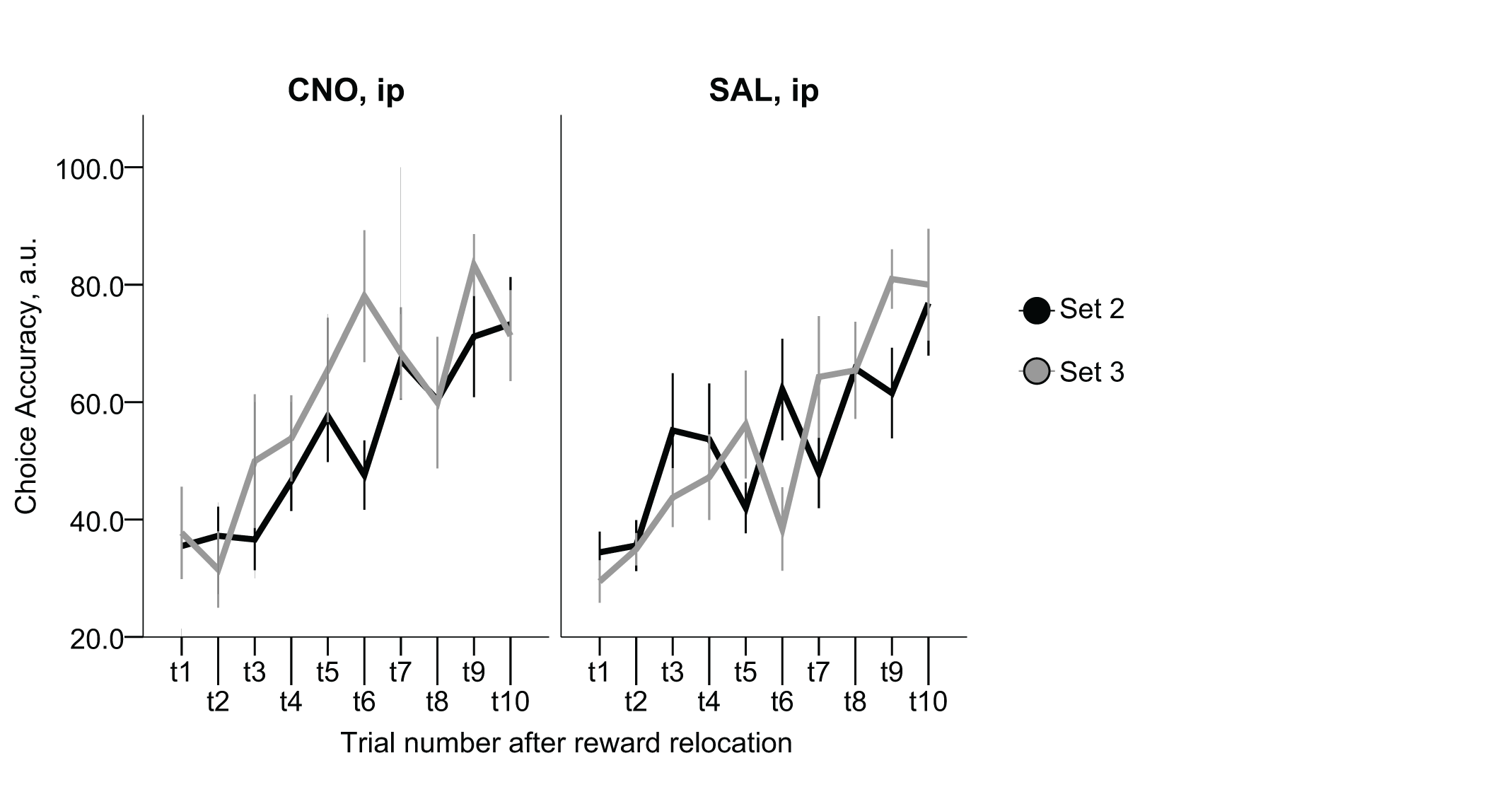

Supplement: Figure 1-2 — Rate of relearning during repeated reward relocations. All animals were administered both CNO and saline and were tested in successive sessions in a counterbalanced order. Choice accuracy improved equally across all trials during the first (Reward Set 2) and second (Reward Set 3) test sessions. Download Figure 1-2, TIF file. [file eneuro-11-ENEURO.0063-24.2024-s004.tif]

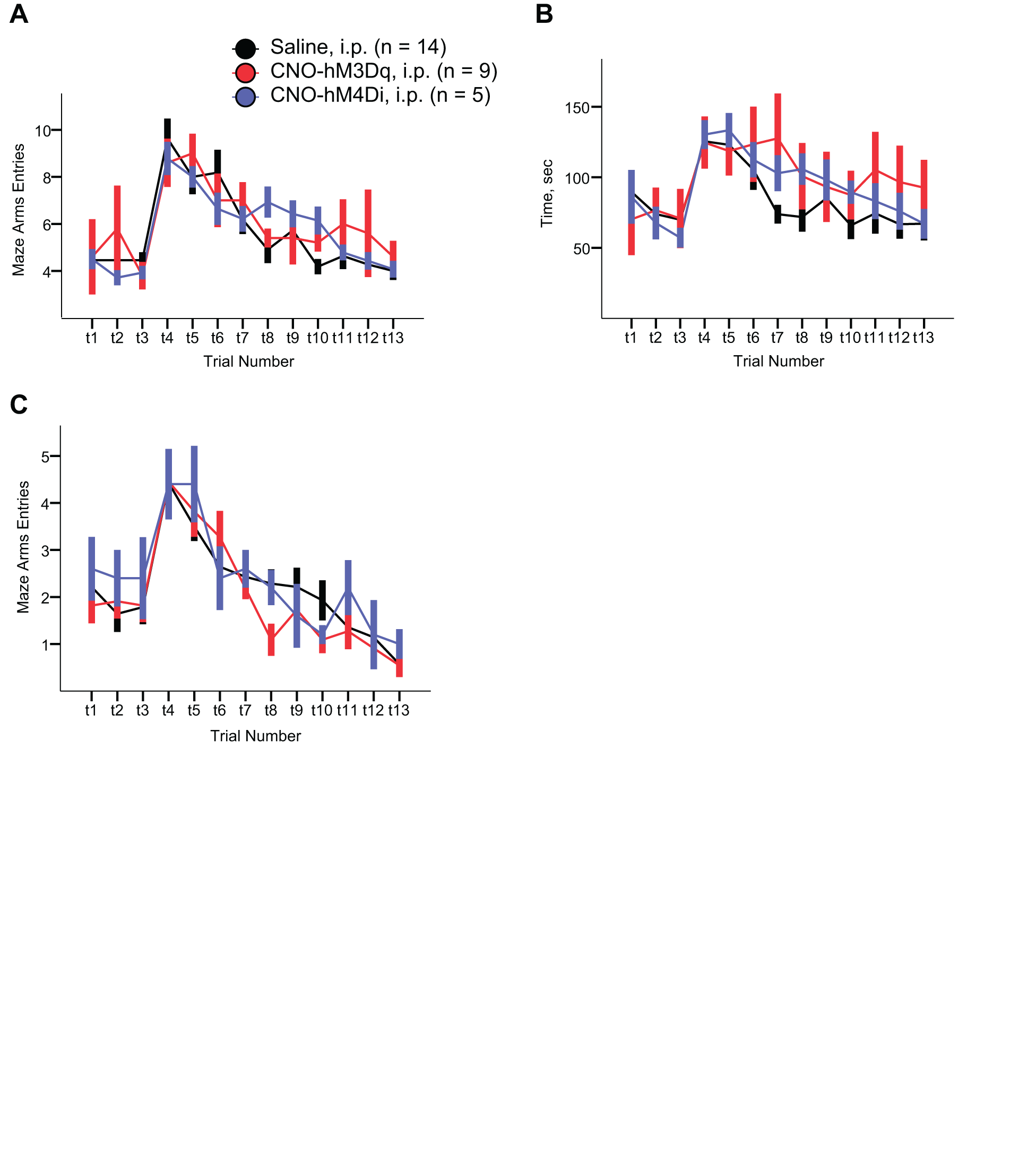

Supplement: Figure 1-3 — The rat behavior during the Reward Relocation Probe. The total number of maze arm entries (A), trial time (B), and entries to previously baited arms (C) are plotted for all trials and experimental groups. The data after saline injection were combined for hM3Dq- and hM4Di-expressing rats. All rats showed equal food-motivated behavior as reflected by the number of maze arm entries in each trial (A). The trial time increased (B) due to rats’ visiting previous reward locations (C). The entries to unbaited maze arms gradually reduced. There was no significant trial x group interaction for any of the variables (Multivariate Pillai's Trace Test; entries: F(18,40) = 1.12, p = 0.372; time: F (6.1,163.9) = 0.86, p = 0.595; unbaited: F(18,40) = 0.78, p = 0.713). Download Figure 1-3, TIF file. [file eneuro-11-ENEURO.0063-24.2024-s005.tif]

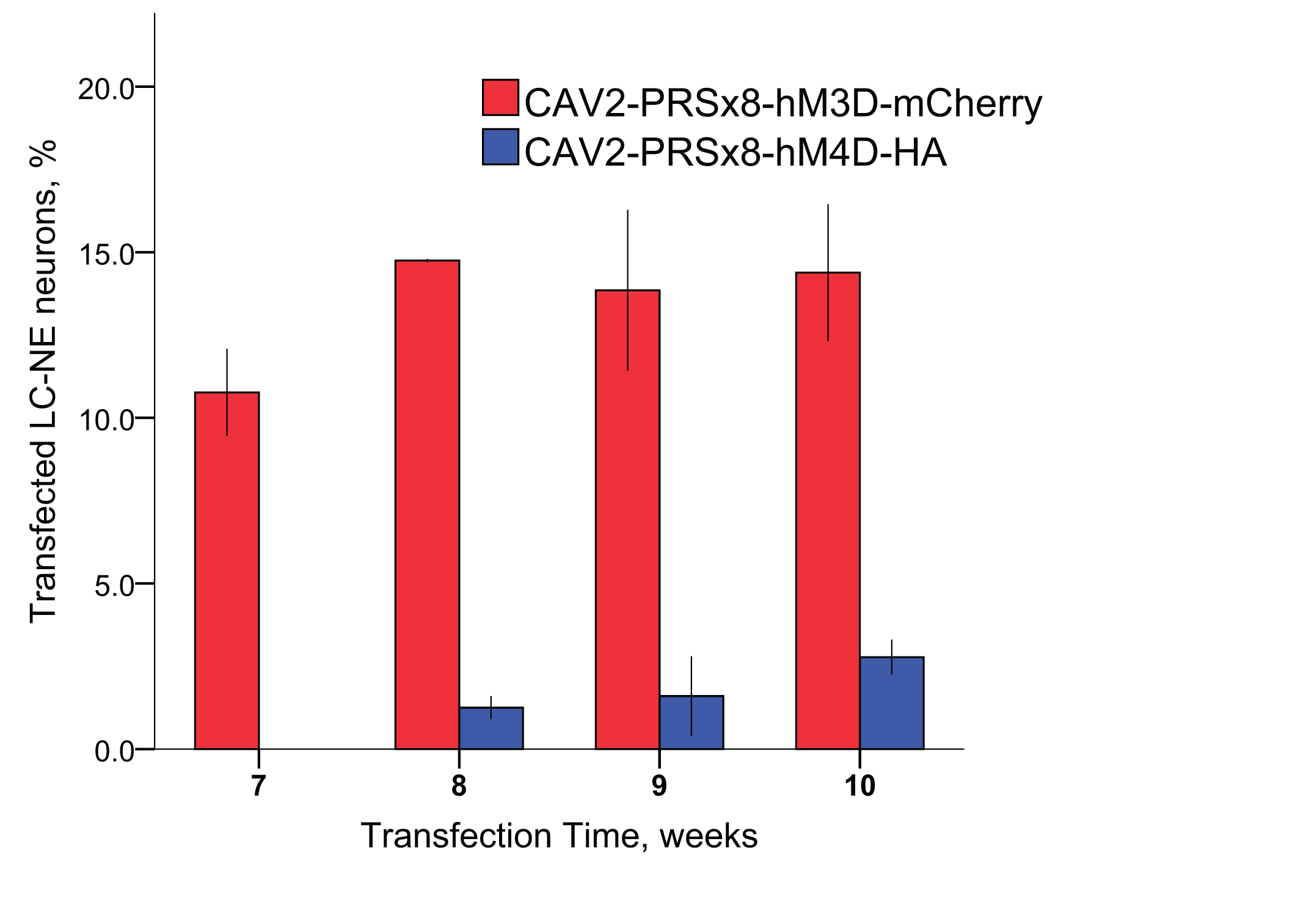

Supplement: Figure 1-4 — DREADD expression level in the ACC-projecting LC neurons did not decay for up to 10 weeks after the virus injection for either viral construct. Download Figure 1-4, TIF file. [file eneuro-11-ENEURO.0063-24.2024-s006.tif]

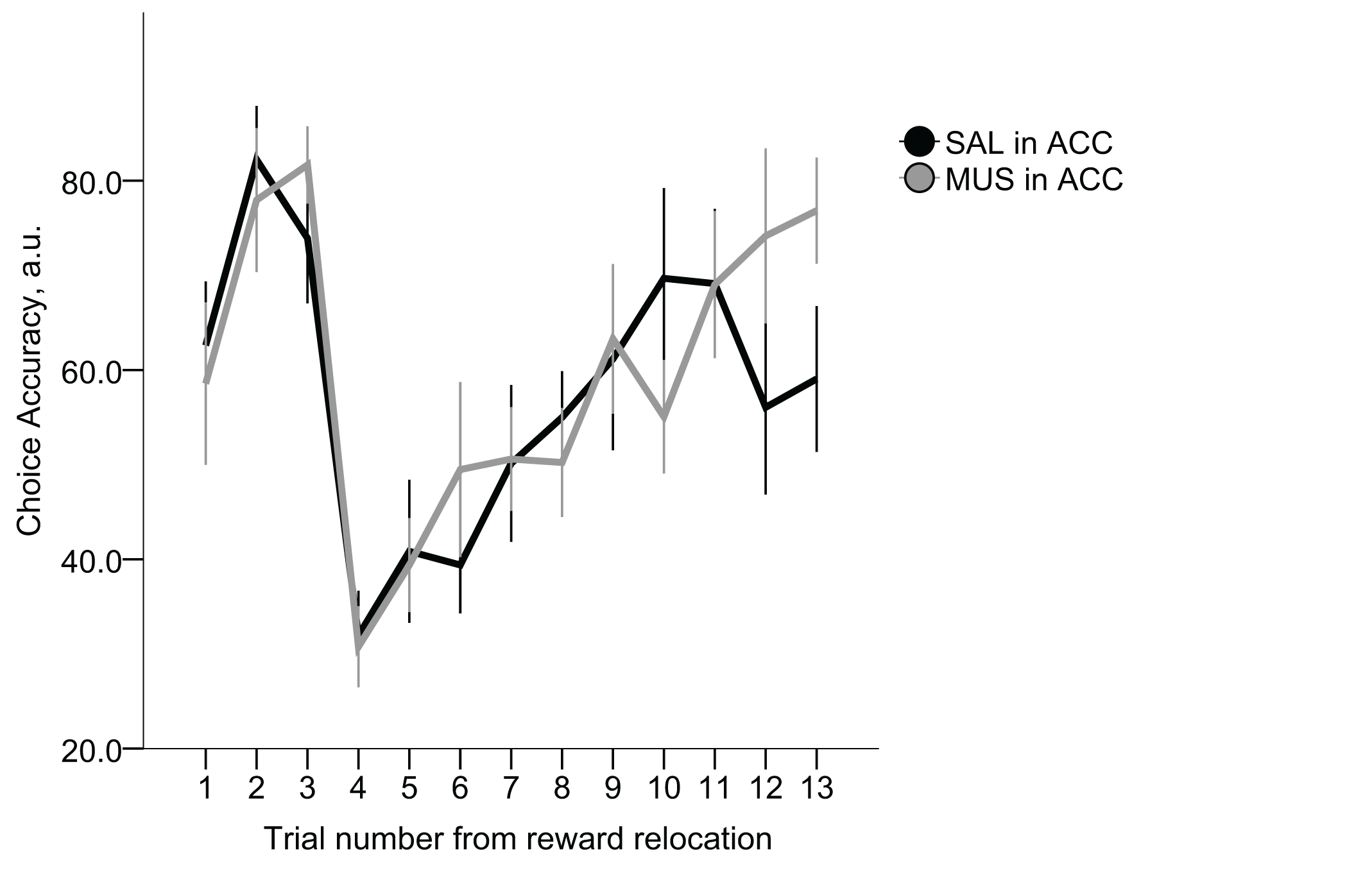

Supplement: Figure 1-5 — The ACC transient inactivation by muscimol did not affect the rate of relearning during the Reward Relocation Probe. A total of 10 muscimol (n = 10 rats) and 12 saline (n = 12 rats) injections were included in the analysis. Download Figure 1-5, TIF file. [file eneuro-11-ENEURO.0063-24.2024-s007.tif]

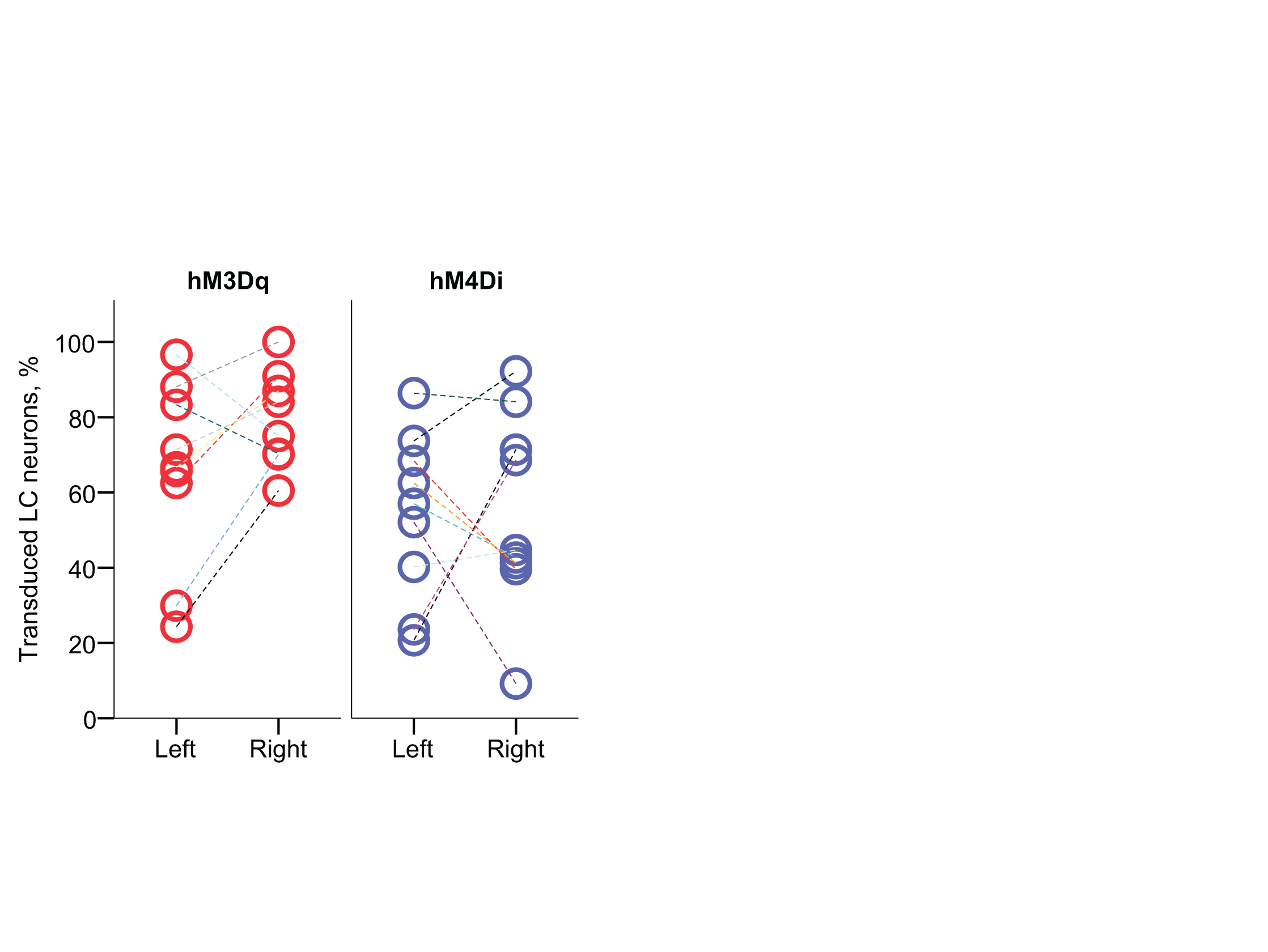

Supplement: Figure 2-1 — The proportion of virus-transduced LC neurons in the left and right hemispheres. Download Figure 2-1, TIF file. [file eneuro-11-ENEURO.0063-24.2024-s008.tif]

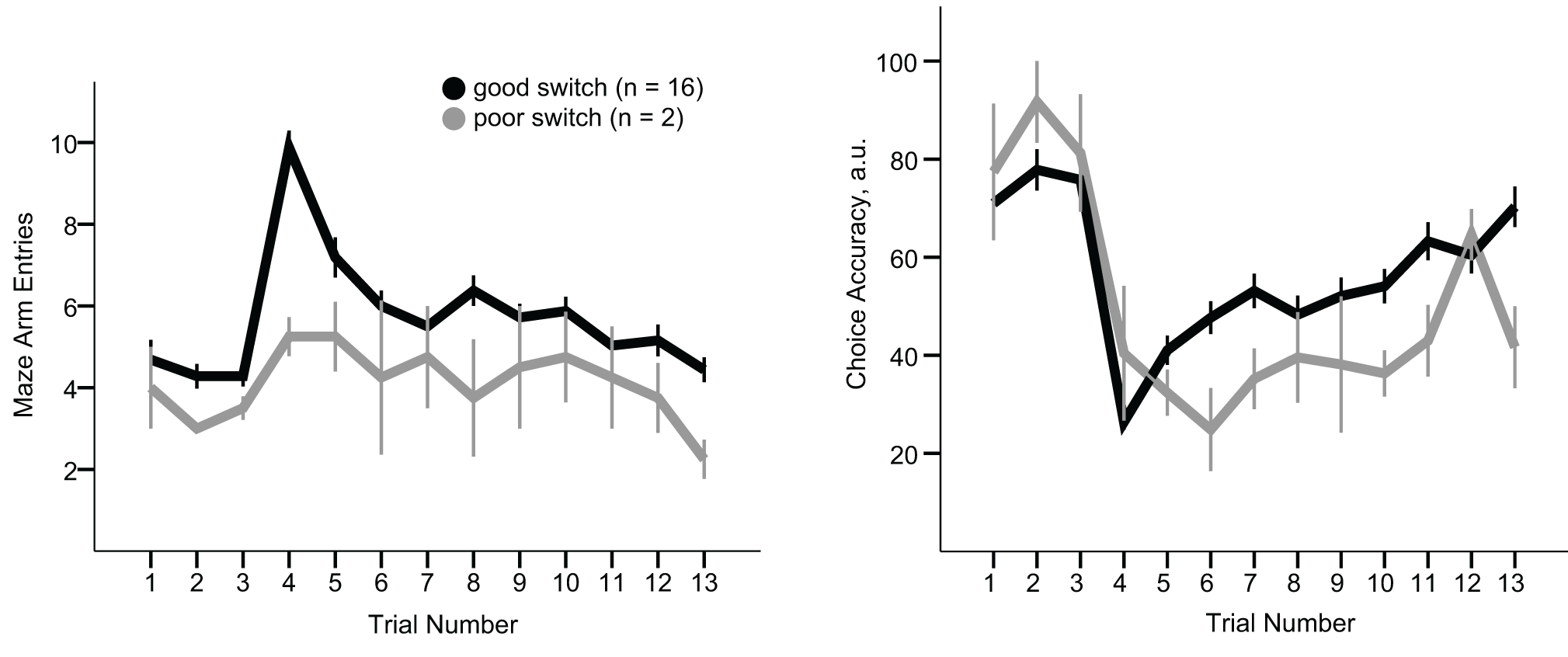

Supplement: Figure 2-2 — The number of maze arm entries (left) and the choice accuracy (right) during the Reward Relocation Probe. Note a robust increase in the number of maze arm entries after the reward relocation on trial 4, except for two rats showing low exploratory activity. The same two rats had the slowest relearning rate (poor switch) despite the good task performance before the reward relocation. The rat behavior did not depend on the drug injection. Download Figure 2-2, TIF file. [file eneuro-11-ENEURO.0063-24.2024-s009.tif]

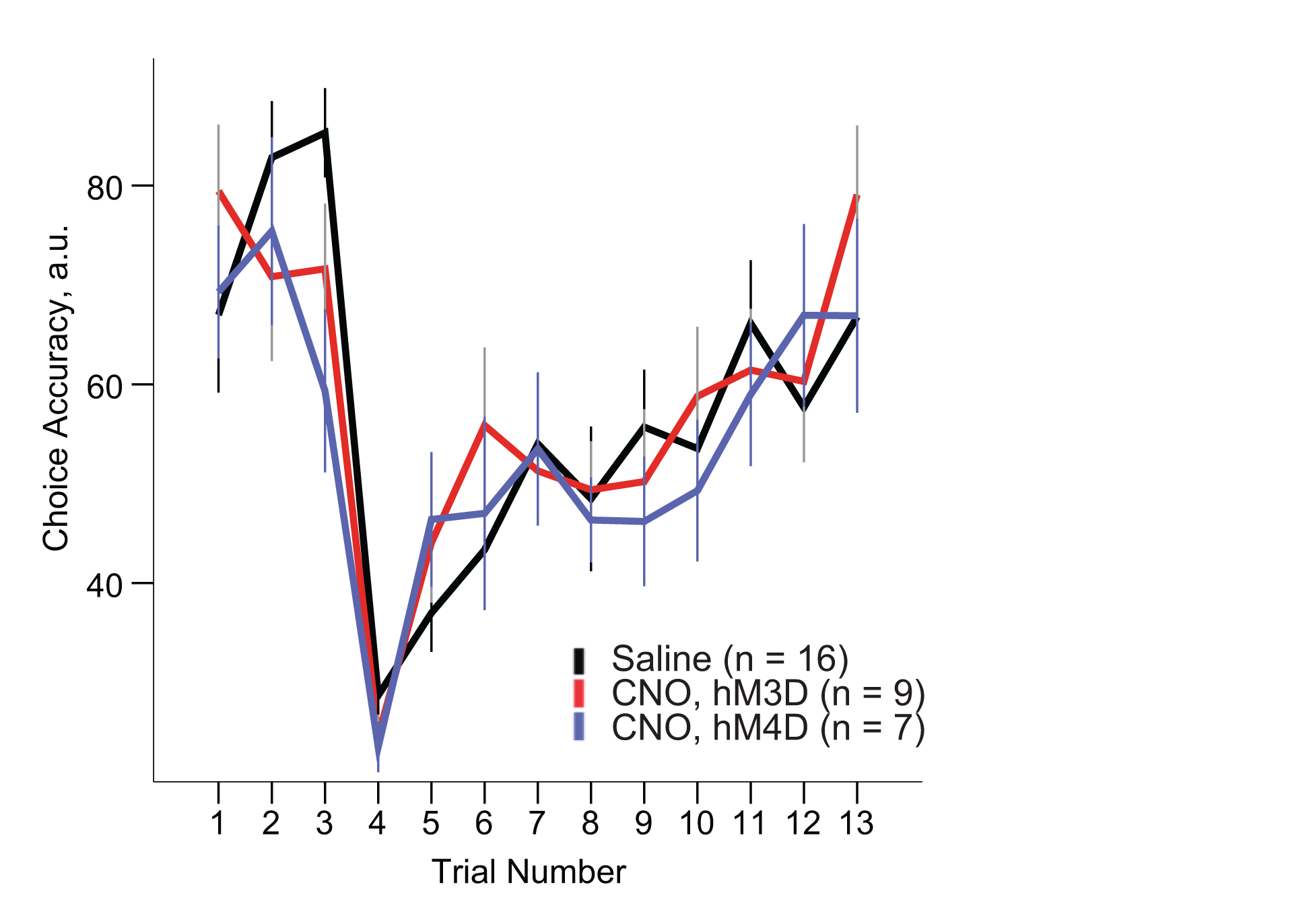

Supplement: Figure 2-3 — The rate of relearning in rats with enhanced (red) or decreased (blue) NE transmission. All animals were administered both CNO and saline and were tested in successive sessions in a counterbalanced order. The choice accuracy improved across trials equally in all groups (F (6.1,21) = 12.72, p < 0.001, Greenhouse-Geisser corrected; trial x group: F(12.2,44) = 0.62, p = 0.89). Download Figure 2-3, TIF file. [file eneuro-11-ENEURO.0063-24.2024-s010.tif]

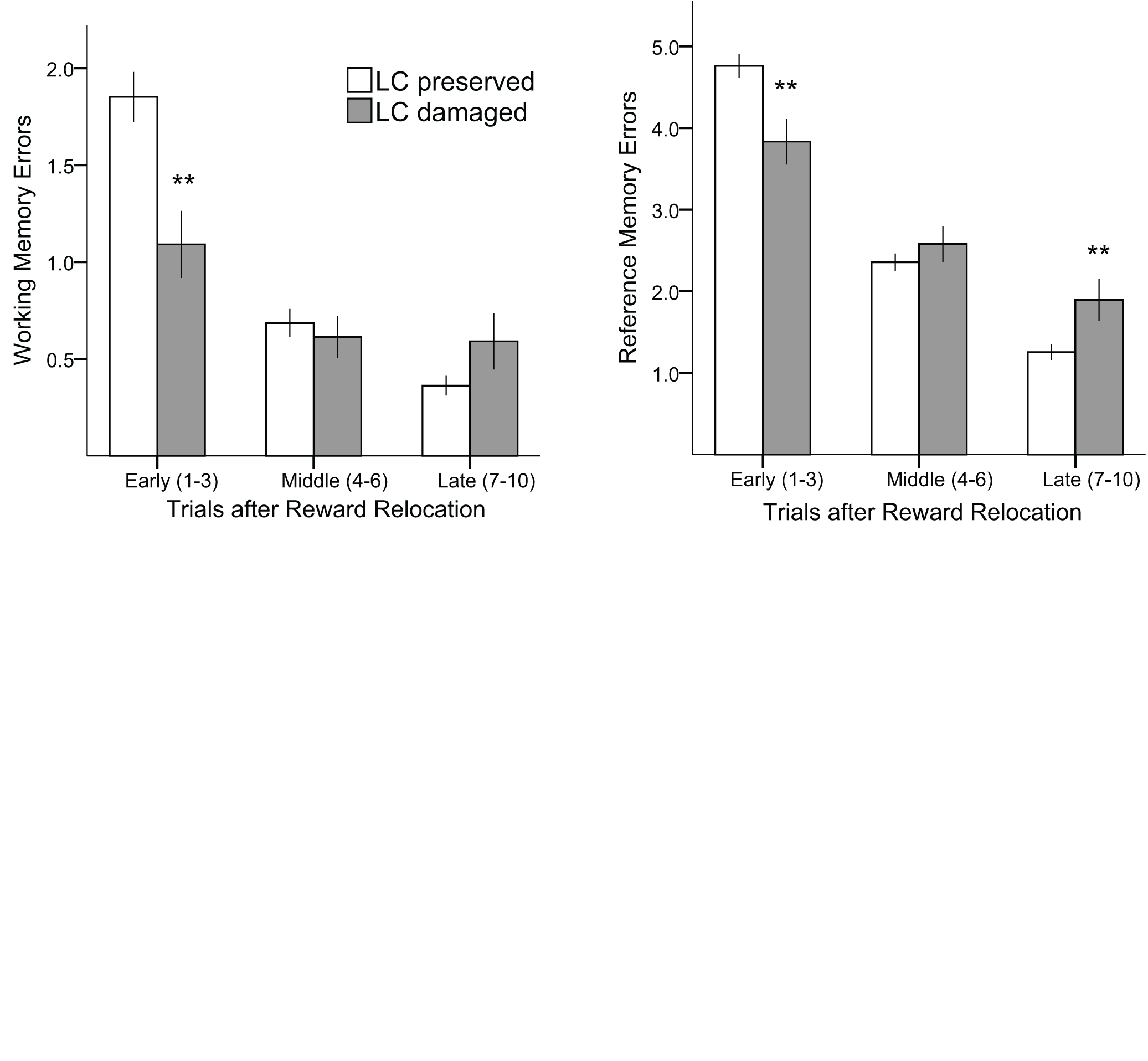

Supplement: Figure 3-1 — The number of working (left) and reference (right) memory errors during the Reward Relocation Probe is shown for the rats with preserved (n = 27) and damaged (n = 8) LC. ** - p < 0.05 (pna-way ANOVA). Download Figure 3-1, TIF file. [file eneuro-11-ENEURO.0063-24.2024-s011.tif]
