# Supplementary material for: The Projection-Specific Noradrenergic Modulation of Perseverative Spatial Behavior in Adult Male Rats
Source: eNeuro. 2024 Aug 15;11(8):ENEURO.0063-24.2024. doi: 10.1523/ENEURO.0063-24.2024 (PMC11334950; doi:10.1523/ENEURO.0063-24.2024)
Supplement: Table 1-1 — Key resources. Download Table 1-1, DOCX file. [file eneuro-11-ENEURO.0063-24.2024-s002.docx]

**Table 1-1**. Key resources

| **Reagent type** | **Designation** | **Source** | **Catalog #** | **Dilution/Dosage** |
| --- | --- | --- | --- | --- |
| Antibody | mouse Dopamine β-hydroxylase (DbH) | Merck | MAB308 | 1:1000 |
| Antibody | Living Colors® DsRed Monoclonal | TaKaRa Bio | 632496 | 1:1000 |
| Antibody | rabbit HA-Tag | Cell Signaling | 3724 | 1:1000 |
| Antibody | Alexa Fluor 488-conjugated donkey anti-mouse IgG | Invitrogen | A32790 | 1:500 |
| Antibody | Cy3 donkey anti-rabbit IgG | Jackson ImmunoResearch | 711-165-152 | 1:200 |
| Fluorescent Dye | Hoechst 33342 | Sigma-Aldrich | 62249 | 1:10000 |
| Chemical compound, drug | CNO | Enzo Life Sciences Inc | BML-NS105-0025 | 1 mg/kg, i.p. |
| Chemical compound, drug | Muscimol | Sigma Aldrich | M1523 | 0.1 μg/μl |
| Viral vector | CAV2-PRS8- hM4D(Gi)-HA-Tag | <https://plateau-igmm.pvm.cnrs.fr/?vector=cav-prs-ha-hm4di> |  | Titre:  1x10^12vp/ml  1.5×10^12vp/mL |
| Viral vector | CAV2-PRS8- hM3D(Gq)-mCherry | <https://plateau-igmm.pvm.cnrs.fr/?vector=cav-prs-hm3dgq-mcherry> |  | Titre:  1x10^12vp/ml  1.5×10^12vp/mL |
| Green fluorescent RetroBeads | Fluorescent Tracer | LumaFluor | [Green Retrobeads™ IX (100 µl)](https://lumafluor.com/shop/ols/products/xn-green-retrobeads-ix-100-l-eib3381v) |  |
